# Genome-wide analysis and expression profile of the bZIP transcription factor gene family in grapevine (*Vitis vinifera*)

**DOI:** 10.1186/1471-2164-15-281

**Published:** 2014-04-13

**Authors:** Jinyi Liu, Nana Chen, Fei Chen, Bin Cai, Silvia Dal Santo, Giovanni Battista Tornielli, Mario Pezzotti, Zong-Ming (Max) Cheng

**Affiliations:** 1 College of Horticulture, Nanjing Agricultural University, 210095 Nanjing, Jiangsu, China; 2 Dipartimento di Biotecnologie, Università degli Studi di Verona, 37134 Verona, Italy; 3 Department of Plant Sciences, University of Tennessee, 37996 Knoxville, TN, USA

**Keywords:** bZIP transcription factor family, Grapevine, Gene expression, Drought response, Heat stress response

## Abstract

**Background:**

Basic leucine zipper (bZIP) transcription factor gene family is one of the largest and most diverse families in plants. Current studies have shown that the bZIP proteins regulate numerous growth and developmental processes and biotic and abiotic stress responses. Nonetheless, knowledge concerning the specific expression patterns and evolutionary history of plant bZIP family members remains very limited.

**Results:**

We identified 55 bZIP transcription factor-encoding genes in the grapevine (*Vitis vinifera*) genome, and divided them into 10 groups according to the phylogenetic relationship with those in Arabidopsis. The chromosome distribution and the collinearity analyses suggest that expansion of the grapevine bZIP (VvbZIP) transcription factor family was greatly contributed by the segment/chromosomal duplications, which may be associated with the grapevine genome fusion events. Nine intron/exon structural patterns within the bZIP domain and the additional conserved motifs were identified among all VvbZIP proteins, and showed a high group-specificity. The predicted specificities on DNA-binding domains indicated that some highly conserved amino acid residues exist across each major group in the tree of land plant life. The expression patterns of *VvbZIP* genes across the grapevine gene expression atlas, based on microarray technology, suggest that *VvbZIP* genes are involved in grapevine organ development, especially seed development. Expression analysis based on qRT-PCR indicated that *VvbZIP* genes are extensively involved in drought- and heat-responses, with possibly different mechanisms.

**Conclusions:**

The genome-wide identification, chromosome organization, gene structures, evolutionary and expression analyses of grapevine bZIP genes provide an overall insight of this gene family and their potential involvement in growth, development and stress responses. This will facilitate further research on the bZIP gene family regarding their evolutionary history and biological functions.

## Background

Transcription factors (TFs) play important roles in regulation and control many crucial biological processes in plants. Functional characterization of TFs is essential for understanding transcriptional regulatory networks and the biological processes they are involved in. Presently, at least 64 families of transcription factors have been identified in the plant kingdom [1]. The basic leucine zipper (bZIP) transcription factor family is one of the largest and most diverse families [1,2]. The bZIP transcription factors harbor a highly conserved bZIP domain composed of two structural features: a basic region (N-x7-R/K-x9) for sequence-specific DNA binding, and a leucine zipper, which is composed of several heptad repeats of Leu or other bulky hydrophobic amino acids, such as Ile, Val, Phe or Met, for dimerization specificity [2-4]. Outside the bZIP domain, the bZIP transcription factors also contain other conserved domains that function as transcriptional activators. For example, two conserved motives, described as R/KxxS/T and S/TxxD/E, have been confirmed as sites phosphorylated by the Ca^2+^ independent protein kinase and casein kinase II, respectively [5]. Proline-rich, glutamine-rich and acidic domains are also thought to play roles in transcription activation of *bZIP* genes [6].

The bZIP gene family has been comprehensively identified or predicted in several plant genomes. Seventy-five *bZIP* genes have been found in Arabidopsis (*Arabidopsis thaliana*) [4], 89 in rice (*Oryza sativa*) [2], 131 in soybean (*Glycine max*) [6], 92 in Sorghum [7], and 125 in maize (*Zea mays*) [8]. Among all members in all plant species, just a small number of *bZIP* genes have been functionally characterized. Plant bZIP proteins have been found to regulate the integration and development of many organs and tissues, including seed maturation and germination [9,10], floral induction and development [11-18], vascular development [19], embryogenesis [20,21] and photomorphogenesis [22,23]. They have also been found to be involved in responses to a variety of abiotic/biotic stimuli, such as drought [24,25], high salinity [26], heat stress [27], cold stress [28,29], pathogen infection [30], and hormone signaling, such as abscisic acid (ABA) [25,31,32], ethylene [33] and light signaling [34].

Grapevine (*Vitis* spp.) is a popular fruit crop that in cultivated throughout the world and represents one of the most important crops with highly valued products such as juice, liquors and wines. The grapevine (*Vitis vinifera*) genome sequence was released in 2007 [35]. It was suggested that the grapevine genome was composed of three ancestral genomes, and it has retained more ancient imprint of the evolution. However, the notion of grapevine as a “palaeo-hexaploid” organism [35,36] remains to be confirmed. Since *Vitis* is sister to all Rosids (I and II), studying the evolution of a large gene family in grapevine will shed light on our understanding the evolution of other plants [36], and will allow the elucidation of function and divergence of gene families in grapevine. So far, only a limited number of *VvbZIPs* have been isolated and functionally characterized [37]. Among them, two *VvABF*s (homologous of *VvbZIP08* and *VvbZIP45* in our study), isolated from the *V. vinifera* cultivar ‘Thompson Seedless’ , were induced by various abiotic stresses including drought, salinity, cold and the exogenous hormone ABA [38], and *VvbZIP45* corresponds to the very recently described *ABSCISIC ACID RESPONSE ELEMENT-BINDING FACTOR2* (*VvABF2*), involved in ABA-dependent grape berry ripening [39]. Another gene, *VvbZIP23*, which was isolated from the cultivar ‘Mango’ , was induced by a wide spectrum of abiotic stresses, including drought, salt, cold, and application of abscisic acid, methyl viologen, salicylic acid, jasmonic acid, and ethephon [37]. In addition, the transcriptional analysis of late ripening stages of grapevine berry detected one bZIP transcription factor which exhibited lower expression at the harvest stages (TH) than that at the 7-days before harvest stage (TH-7) [40]. The transcriptomic analysis of grape leaves suggested that some VvbZIP transcription factors were involved in stress responses [41].

In the present study, we report the identification of 55 bZIP genes from the grapevine genome, and the analysis of the gene family with a focus on the evolution and divergence after multiple duplications in relation to the grapevine genome fusion. We also analyze gene expression of *VvbZIP* genes in different tissues/organs/developmental stages, as well as in response to stresses. Our results provide a prospective for the evolutionary history and general biological involvement of the grapevine bZIP transcription factor family.

## Results

### Identification and nomenclature assignment of bZIP transcription factors in grapevine

In order to run a complete search for *VvbZIP* genes in the grapevine genome, all annotated proteins of the latest version (V1) of the grapevine genome from CRIBI (http://genomes.cribi.unipd.it/) were considered. The Hidden Markov Model (HMM) profile of the bZIP domain (PF00170) (http://pfam.sanger.ac.uk/) was then employed as a query to search the database using the program HMMER3.0 with the default E-value. After determining the integrity of the bZIP domain using the online program SMART (http://smart.embl-heidelberg.de/) with an E-value < 0.1 and sequence alignment, 55 non-redundant genes were assigned as grapevine bZIP genes, and named from *VvbZIP01* to *VvbZIP55* based on the coordinate order on grapevine chromosomes from top to bottom. The nomenclature and corresponding information are listed in Table 1.

**Table 1:** **The identified *VvbZIP *** **genes and their related information**

**Gene name**	**Gene ID**	**Group**	**Chr.**	**Locus**		**Chain**	**Protein Length**	**Amount of intron**	**Intron pattern within bZIP domian**	**AtbZIP Ortholog (Syntenic orthologs were denoted by overstriking and italics)**
*VvbZIP01*	VIT_01s0011g03230	J	1	2941266	2946275	+	453	10	*b*	***46***, 20, 45, 26, 65, 21, 57, 47, 22, 50
*VvbZIP02*	VIT_01s0010g00930	C	1	16444033	16444682	-	195	0	*h*	42, ***58***, 48, 43, 3, ***8***, 70, 2, 44, 53, 7, 11, 41, 5, 68, 6, 16, 4
*VvbZIP03*	VIT_02s0025g01020	D	2	989255	996186	+	398	10	*a*	***55(GBF3)***, ***54(GBF2)***, 16, 68, 41
*VvbZIP04*	VIT_02s0012g02250	D	2	9137736	9153313	-	413	11	*a*	55, 54, ***16***, ***68***, 41, 44, 42, 43, 58, 3, 70, 8, 48
*VvbZIP05*	VIT_03s0038g00860	F	3	689247	693308	+	676	5	*c*	***29***,***30***, 18, 52, 69, 51, 59, 31, 33, 61, 34, 74
*VvbZIP06*	VIT_03s0038g02420	I	3	1679507	1683223	+	271	1	*h*	***23***, ***19***, 24
*VvbZIP07*	VIT_03s0038g04450	C	3	3227416	3228660	-	157	0	*h*	44, 11, 2, 53, 48, 3, 42, 43, 58, 8, 1, 6, 7, 5, 68, 70
*VvbZIP08*	VIT_03s0063g00310	A	3	3887073	3890514	-	409	4	*a*	***37(ABF3)***, 36, ***38(ABF4/AREB2)***, ***35(ABF1)***, 15, 66, 12, 67, 39, 14, 13, 40
*VvbZIP09*	VIT_04s0008g02750	B	4	2284121	2287115	-	349	5	*d*	***9***, 63, 10, 2, 53, 42, 25, 44, 58, 3, 48, 68, 11
*VvbZIP10*	VIT_04s0008g05210	G	4	4716191	4719252	+	169	3	*f*	***56(HY5)***, 64
*VvbZIP11*	VIT_04s0069g01150	A	4	9841491	9842587	+	57	1	*a*	66, 12, 37, 36, 35, 38, 39
*VvbZIP12*	VIT_04s0023g01360	D	4	17832626	17863952	+	364	10	*a*	***41***, 16, 68, 55, 54, 42, 70, 3, 58, 43, 48, 8, 44, 18
*VvbZIP13*	VIT_04s0023g02430	C	4	18983616	18984767	+	249	0	*h*	44, 11, 2, 53, 48, 42, 58, 3, 43, 8, 7, 6, 1, 5
*VvbZIP14*	VIT_05s0077g01140	C	5	878161	879142	-	154	0	*h*	53, 2, 44, 11, 58, 3, 5, 48, 7, 1
*VvbZIP15*	VIT_05s0020g01090	G	5	2860048	2863747	+	210	3	*h*	***64(HYH)***, 56
*VvbZIP16*	VIT_05s0102g01120	F	5	23226450	23231749	-	425	3	*c*	69, 59, 18, 52, 51, 29, 30, 61, 33, 31, 34, 74
*VvbZIP17*	VIT_06s0004g08070	F	6	8835674	8841051	-	342	3	*c*	***18***,***52***, 59, 69, 51, 29, 30, 33, 61, 31, 74, 34
*VvbZIP18*	VIT_06s0009g01790	A	6	13809459	13815625	-	248	2	*a*	66, 12, 35, 38, 36, 67, 39, 40, 14, 13, 37, 15
*VvbZIP19*	VIT_06s0080g00340	A	6	20262007	20263157	+	324	2	*a*	***67(DPBF2)***, 39, 66, 12, 35, 36, 38, 15, 37, 40
*VvbZIP20*	VIT_06s0080g00360	J	6	20304714	20311349	-	500	10	*g*	***21***, 65, 45, 20, 26, 46, 22, 50, 47, 57
*VvbZIP21*	VIT_07s0141g00170	B	7	116657	126811	-	452	5	*d*	25, 10, 63, 9
*VvbZIP22*	VIT_07s0005g01450	C	7	3987031	3988120	+	145	0	*h*	***53***, 44, 2, 11, 42, 3, 6, 48, 7, 58, 43, 8, 16, 1, 68
*VvbZIP23*	VIT_07s0031g01320	J	7	17404970	17410595	+	349	7	*b*	47, ***57***, 50. 22, 20, 45, 26, 21, 46, 65
*VvbZIP24*	VIT_08s0040g00870	H	8	11857207	11860728	-	819	1	*h*	17, 28, ***49***
*VvbZIP25*	VIT_08s0007g03420	A	8	17372100	17378057	-	400	0	*a*	***39(ABI5)***, 35, 67, 66, 38, 37, 36, 12, 13, 15, 40, 14
*VvbZIP26*	VIT_08s0007g03640	UC	8	17579864	17586189	+	496	5	*a*	62
*VvbZIP27*	VIT_08s0007g05170	J	8	19088004	19098613	-	451	10	*b*	45, 20, 26, 46, 21, 65, 57, 50, 22, 47
*VvbZIP28*	VIT_08s0007g06160	J	8	19963578	19968190	-	491	10	*b*	***65***, 21, 45, 20, 26, 46, 22, 57, 50, 47
*VvbZIP29*	VIT_12s0028g02590	E	12	3366293	3367561	-	295	3	*e*	61, 34, 51, 69, 18, 52, 59, 30, 29, 72, 33, 31
*VvbZIP30*	VIT_12s0055g00420	A	12	13276799	13300251	+	299	3	*a*	13, 40, 12, 66, 67, 35, 37, 38, 36, 39, 14
*VvbZIP31*	VIT_12s0034g00110	A	12	15485471	15491224	+	99	3	*a*	66, 12, 37, 36, 35, 38, 39
*VvbZIP32*	VIT_12s0035g00620	E	12	20038502	20045379	+	374	3	*e*	61, 34, 69, 59, 18, 51, 29, 52, 30
*VvbZIP33*	VIT_13s0067g02900	F	13	1562503	1569804	+	359	3	*c*	***18***, 52, 59, 69, 51, 29, 30, 33, 61, 31, 34, 74, 72
*VvbZIP34*	VIT_13s0175g00120	A	13	15049338	15072969	+	325	2	*a*	66, 12, 35, 67, 38, 37, 36, 39, 15, 14
*VvbZIP35*	VIT_13s0084g00660	J	13	19780668	19805654	-	469	10	*b*	45, 26, 20, 46, 21, 65, 57, 22, 50, 47
*VvbZIP36*	VIT_13s0158g00380	I	13	21283084	21284766	-	286	1	*h*	23, 19, 24
*VvbZIP37*	VIT_14s0060g01210	C	14	959578	961198	-	145	0	*h*	53, 11, 2, 44, 1, 48, 58, 3, 8, 7, 42, 43, 70
*VvbZIP38*	VIT_14s0030g02200	B	14	7402807	7408597	+	423	5	*d*	63. 10. 25. 9
*VvbZIP39*	VIT_14s0083g00700	C	14	22839619	22840360	-	198	0	*h*	***42***,***43***, 58, ***3***,48, 8, 70, 44, 2, 53, 6, 11, 41, 7, 5, 68
*VvbZIP40*	VIT_15s0046g01440	D	15	18416554	18423462	-	430	11	*a*	***55(GBF3)***, ***54(GBF2)***, 16, 68, 41, 3, 42, 43, 70, 58
*VvbZIP41*	VIT_18s0122g00500	UC	18	420616	423564	+	322	2	*f*	***60***
*VvbZIP42*	VIT_18s0001g04470	J	18	3861987	3867762	-	363	7	*b*	57, 47, 50, ***22***, 26, 20, 65, 45, 21, 46
*VvbZIP43*	VIT_18s0001g04500	A	18	3875315	3878509	+	311	2	*a*	12, 66, 35, 36, 39, 38, 37, 14, 15, 67, 40, 13
*VvbZIP44*	VIT_18s0001g08710	C	18	7185268	7185664	-	81	0	*h*	7, 6, 4, 5, 2, 3
*VvbZIP45*	VIT_18s0001g10450	A	18	8761978	8769742	-	447	3	*a*	***36(ABF2/AREB1)***, 35, ***37(ABF3)***, 38, 66, 12, 39, ***15***, 67
*VvbZIP46*	VIT_18s0001g12120	D	18	10309606	10313842	+	396	10	*a*	41, 16, 68, 55, 54, 43, 58, 42, 3, 48, 70, 8
*VvbZIP47*	VIT_18s0001g13040	C	18	11138196	11139020	+	154	0	*h*	44, 11, 2, 53, 48, 3, 42, 58, 43, 1, 7, 6, 8, 5
*VvbZIP48*	VIT_18s0001g13740	F	18	11745490	11746413	-	187	3	*c*	30, 29, 51, 69, 52, 18, 59, 33, 31, 61, 34
*VvbZIP49*	VIT_18s0001g14890	A	18	12936974	12937903	-	214	2	*a*	***14(FD)***, 66, 12, 37, 36, 39, 38, 35, ***27***
*VvbZIP50*	VIT_18s0076g00330	F	18	16209694	16218061	+	350	3	*c*	51, 18, 69, 59, 52, 29, 30, 31, 61, 33, 74, 34
*VvbZIP51*	VIT_18s0072g00470	A	18	19680318	19687522	+	275	3	*a*	13, 40, 66, 12, 39, 36
*VvbZIP52*	VIT_19s0014g01780	E	19	1945207	1947498	-	309	3	*e*	61, 34, 51, 18, 52, 69, 59, 30, 29, 72, 33, 31
*VvbZIP53*	VIT_19s0015g01020	A	19	9101591	9112651	-	251	2	*g*	36, 12
*VvbZIP54*	VIT_18s0001g03010	E	18_random	3068050	3070292	-	272	3	*e*	61, 34, 69, 51, 59
*VvbZIP55*	VIT_00s0541g00020	C	Un	31593865	31594595	-	194	0	*h*	6, 5, 7, 44, 42, 58, 2, 3, 11, 48, 53, 43, 70, 8, 1, 4

Note: AtbZIP Orthologs were identified by local BLAST search with E-value < 1E-10 and the identification of syntenic orthologs (highlight with italics) was performed by using the software MCScanX.

### Phylogenetic analysis and classification of the grapevine bZIP gene family

**Figure 1:**
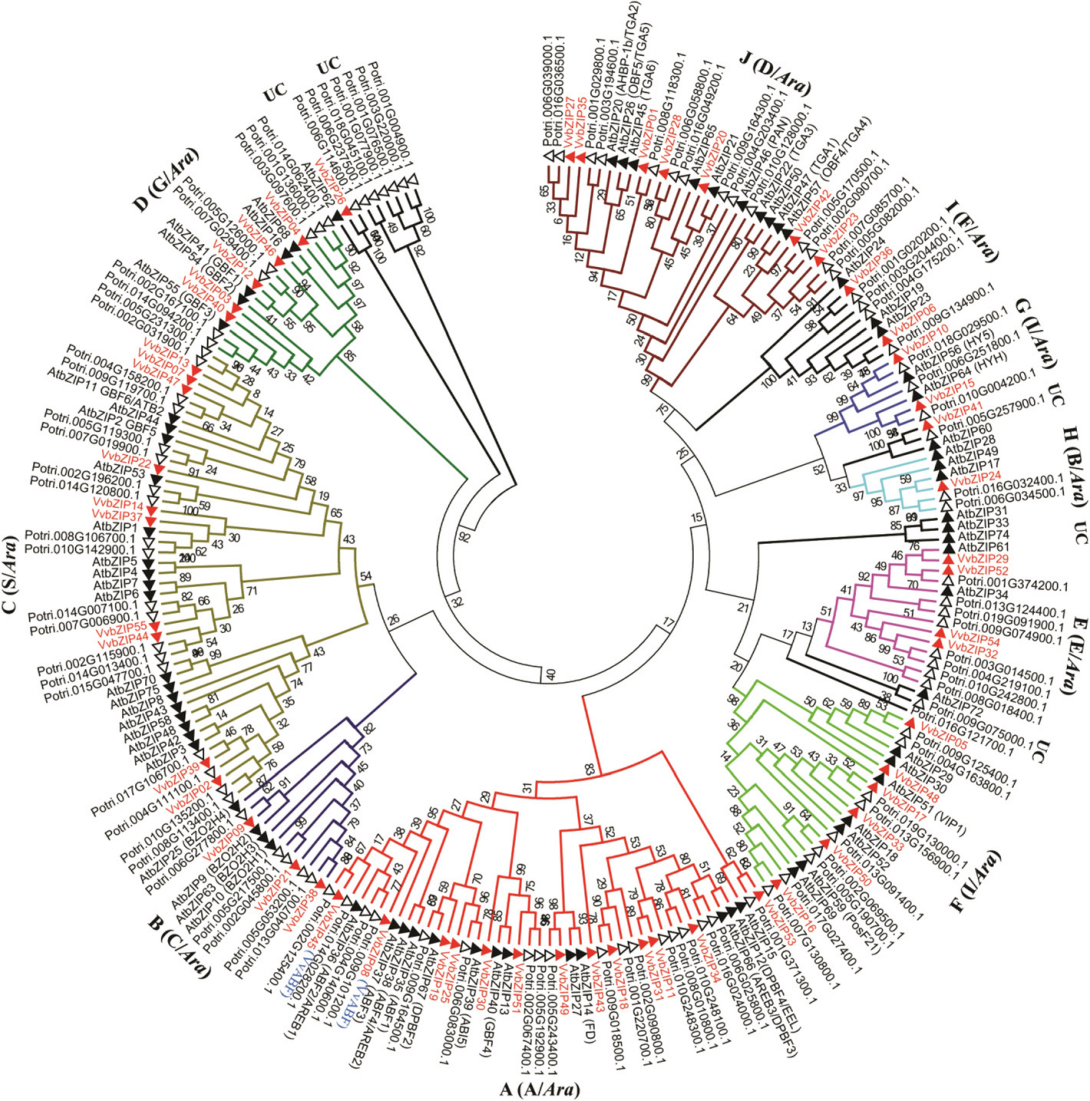
**The phylogenetic tree of grapevine and Arabidopsis bZIP genes.** The 55 grapevine, 72 Arabidopsis and 99 poplar bZIP domain protein sequences were aligned by Clustal X 1.83 and the phylogenetic tree was constructed using MGA5.0 by the Neighbor-Joining (NJ) method. The Bootstrap value was 1,000 replicates. The gene names of the characterized bZIP genes are shown in parenthesis after the gene numbers, and which were referred to previous research [2,4]. A(A/*Ara*), B(C/*Ara*) and so on is presented as the subfamily names of bZIP gene family in grapevine (capital letter outside the parenthesis) and Arabidopsis (capital letter in parenthesis and *Ara* represent for Arabidopsis), UC, unclassed genes or clades. The colored branch indicates the different subfamily and the black represent for UC.

To investigate the phylogenetic relationship of the bZIP gene families in grapevine and Arabidopsis, the amino acid sequences of the bZIP domain of the 55 proteins from grapevine (*Vitis vinifera*), 72 from Arabidopsis (*Arabidopsis thaliana*) (three genes were no longer supported by their annotations comparing to Jakoby et al. [4]) and 99 from poplar (*Populus trichocarpa*) (data from Phytozome v9.1) were used to construct a phylogenetic tree. Three methods, Neighbor-Joining (NJ), Minimal Evolution (ME) and Maximum Parsimony (MP), yielded nearly identical phylogenetic trees (data not shown), therefore, only the NJ tree was used for further analyses (Figure 1). Based on the phylogenetic tree, the *VvbZIP*s can generally be divided into the same 10 subgroups as those in Arabidopsis. However, *AtbZIP62*/*VvbZIP26/Potri.006G114600.1*, *AtbZIP60*/*VvbZIP41/Potri.005G257900.1*, and *AtbZIP72/Potri.009G075000.1* (UC, Figure 1) form three small unique clades in the phylogenetic tree and may have had independent evolutionary trajectories from others clades. In addition, *AtbZIP74*, *AtbZIP33* and *AtbZIP31* and *Potri.006G237500.1/Potri.018G045100.1/Potri.001G077900.1/Potri.001G076500.1/Potri.003G220000.1/Potri.001G004900.1*, were distinguished from other bZIP genes as they formed individual clades without any other bZIP genes, suggesting that these individual clades may be specific to Arabidopsis and poplar, respectively. This result, different from the former classification in Arabidopsis [4], was supported by Nijhawan et al. and Wei et al. [2,8] and sustained by the analyses of the gene structures, conserved motifs and DNA-binding site specificity of VvbZIP transcription factors in the present work.

### Chromosomal location and expansion patterns of *VvbZIP* genes

**Figure 2:**
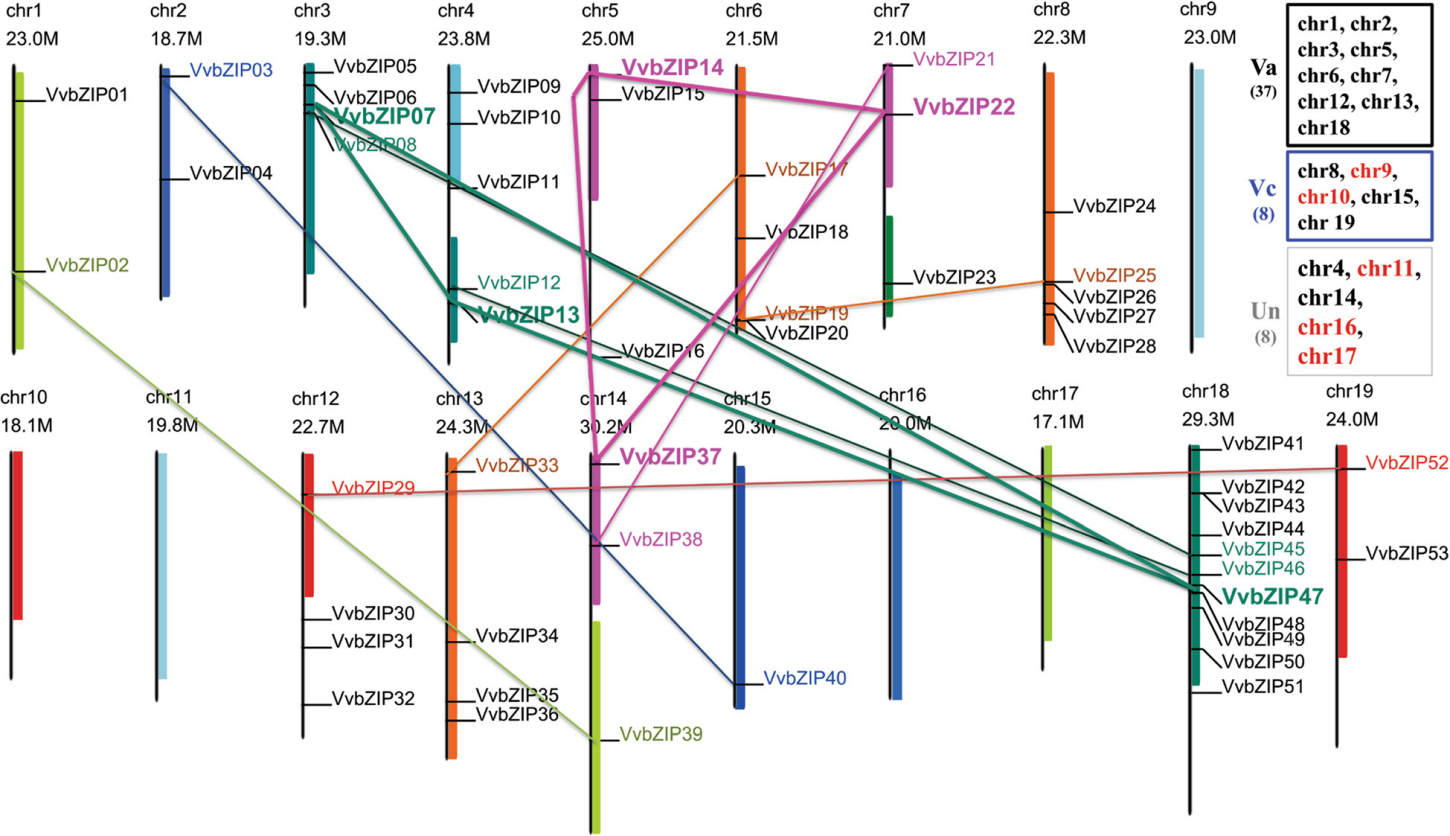
**Distributions of *VvbZIP *genes in grapevine Chromosomes.** Fifty-three *VvbZIP* genes were mapped to the 14 linkage groups. *VvbZIP54* was located on 18 random chromosomes and *VvbZIP55* was assigned to the Un chromosome. Five chromosomes (chr9, 10, 11, 16 and 17) have no grapevine bZIP gene, The schematic representation of paralogous regions was adapted from Jaillon et al., with the same color [35]. Acording to the former hypothesis [36], the ancestral hexaploid genome of *V. vinifera* was derived from an allopolyploidization of two ancestral genomes: Va and Vc, which are denoted on the right of the figure, and Un is unassigned chromosomes. The chr names in red underline the chromosomes that harbored no bZIP genes. The gene names with collinearity were performed with the some color of chromosome paralogous region.

The Vitaceae evolved about 60 million years ago (MYA) and is the earliest diverging lineage of rosids [36,42]. The extant grapevine genome was considered to be formed by fusion of three ancestral genomes following post-fusion shuffling [36] which has not undergone recent genome duplication following the γ WGD. The “palaeo-hexaploid” nature of the genome is supported by many triplicate blocks in the genome, as indicated by the same colors of homeologous blocks adapted from Jaillon et al. [35], and it was hypothesized that the ancestral hexaploid genome of *V. vinifera* was derived from an allopolyploidization of two ancestral genomes, which were named as Va and Vc, respectively [36]. Chromosome triplets consist of two Va and one Vc chromosomes, as expected from the tetraploid and diploid conditions of the two component genomes [36]. To search for imprints of the grapevine genome evolution from the perspective of gene families, the location of each *VvbZIP* gene was given a diagrammatic representation based on the annotation of the 12× V1 assembly grapevine genome available at CRIBI (Figure 2). The results showed that the distribution of *VvbZIP* genes is not even. *VvbZIP54* and *VvbZIP55* are located on the 18 Random and Un chromosome, respectively. Among the other 53 *VvbZIP* genes, 11 (20.75%) genes were situated on chromosome 18; 5 (9.43%) *VvbZIP* genes were each located in the chromosome 4 and 8; 4 *VvbZIP* genes positioned each in chromosomes 3, 6, 12 and 13; 3 *VvbZIP* genes located each in chromosomes 5, 7 and 14; 2 VvbZIP genes each in chromosomes 1, 2 and 19; only one gene in chromosome 15; and no *VvbZIP* genes are located in chromosomes 9, 10, 11, 16 and 17.

Furthermore, we analyzed the tandem arrangement and the collinearity by using MCScanX program and mapped the genes to chromosomal locations (Figure 2), and found 14 homologous blocks containing bZIP genes with collinearity that may have resulted from genome fusion or WGD and none of the *VvbZIP* genes was found to be arranged in tandem. Interestingly, all of those collinearity events occurred within same homologous blocks, and only two triplicate bZIP genes, *VvbZIP7/13/47* and *VvbZIP14/22/37*, were identified, indicating that the expansion patterns of bZIP gene family partially support the triplicate state of the grapevine genome and combined with further genome fusion or WGD events. Further, the rest of the collinearity events were just found within two of the homologous triplets, and the third homologous triplet, such as chromosome 9, 10, 11, 16 and 17, mostly harbored no *VvbZIP* genes, which seem to belong to Vc genome of grapevine described by Malacarne et al. [36], supposing that *VvbZIP* genes in the heterogenous Vc genome may have undergone extensive loss and shuffling after genome fusion.

### Gene structure of *VvbZIP* genes

**Figure 3:**
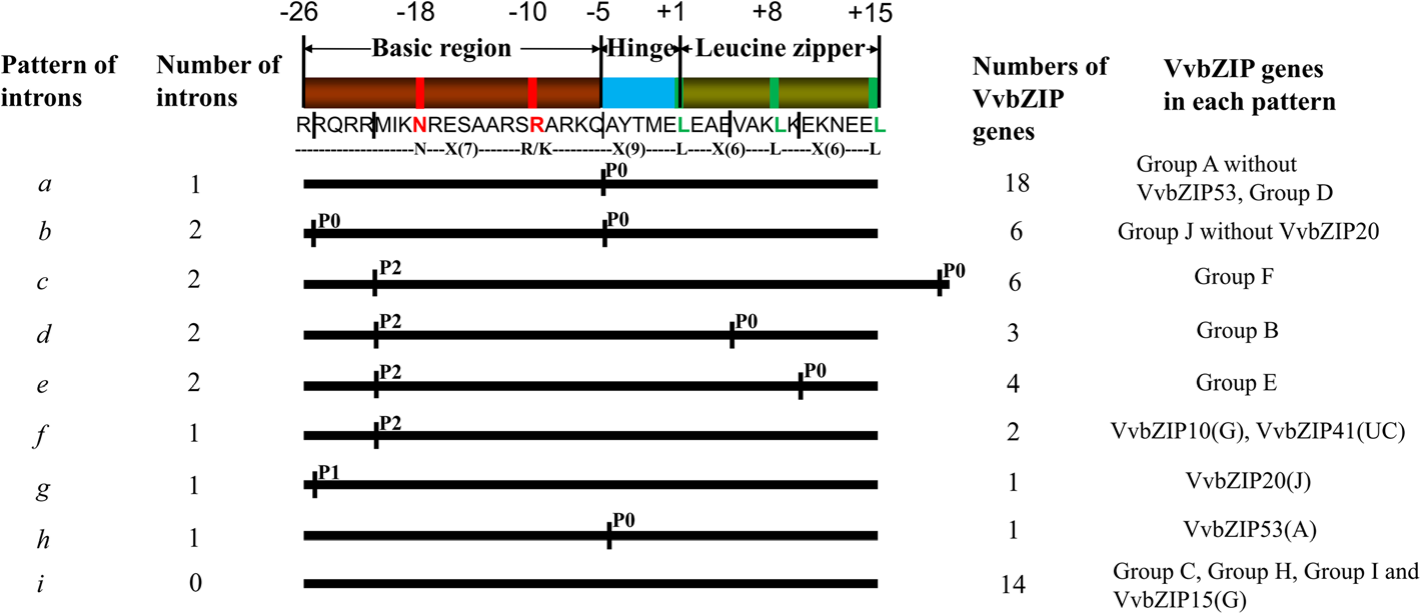
**A schematic diagram of intron patterns in the grapevine bZIP gene family.** The primary structure of the bZIP domain is shown at the top of the picture. The basic region, hinge and leucine zipper were shaded in crimson, blue and green, respectively. The highly conserved residues are highlighted with red and light green boxes. An example of the consensus sequence is given below, and the corresponding positions are shown at the top of the bar [45]. Leucines are sometimes replaced by Ile, Val, Phe or Met. The black vertical lines in the bar indicated the intron position within the bZIP domain and P0, P1 and P2 stand for the intron phase 0, phase 1 and phase 2, respectively. The details of intron position within bZIP domain of the *VvbZIP* genes were shown at Additional file 2.

Since the intron/exon organizations and intron types and numbers are typical imprints of the evolution within some gene families [2,43,44], the *VvbZIP* gene structures were examined to obtain further insight into their evolutionary trajectory. The results show that exon/intron structures of *VvbZIPs* were highly conserved within subfamilies. Genes that clustered together generally possessed a similar gene structure, especially the exon number and length (Additional file 1a). Interestingly, no intron containing genes were found in group C, which accounted for 18.2% (10/55) of the total *VvbZIP* genes. Furthermore, the number of exons in each group (Additional file 1b) was uneven. The numbers of exons in group B, C, E, G and I were much more conserved than that in those in the group A (varying from one to 5). Furthermore, the positions and phases of introns in the bZIP domain (composing of basic region, hinge and leucine zipper) were analyzed (Figure 3). Nine intron/exon structural patterns in the *VvbZIP* gene family were identified based on the intron position, number and splicing phase (details were represented in Additional file 2), similar to those in rice and maize [2,8]. We named these patterns as *a, b, c, d, e, f, g, h* and *i*. The patterns *a, b* and *i* in *VvbZIP* correspond to the patterns *a, b* and *f* in maize and the patterns *b, c* and *g* in rice, respectively. The patterns *c, d, e* and *f* in *VvbZIP* jointly correspond to the pattern *c* in maize or the pattern *a* in rice, because they have the same position of intron in the basic and hinge regions.

In addition, each subfamily of *VvbZIPs* mostly shared the same intron/exon structural pattern. As illustrated in Figure 3, Pattern *a* has just one intron in phase 0 (P0) in the leucine zipper which inserts between the amino acids Gln and Ala, includes members of group A without *VvbZIP20* and all genes of the group D. Pattern *b* has two introns in phase 0 (P0) in the basic and hinge regions, respectively, and includes the genes of group J without *VvbZIP20*. The pattern c, *d, e* and *f* share a common intron insert with phase 2 (P2) in the basic region, but for each pattern, differences in intron inserts exist in the leucine zipper region, Pattern *c, d* and *e* have another intron insert in phase 0 (P0) in the leucine zipper region at a different position, whereas the pattern *f* has no intron insert in the leucine zipper region thus different from *c, d* and *e;* and the pattern *c*, *d* and *e* are completely corresponded to the group F, B, E, respectively. Pattern *g* has only one intron in phase 1 (P1) in the basic region and pattern *h* has one intron in phase 0 (P0) in the leucine zipper region; both patterns *g* and *h* contained just one member of *VvbZIP* gene each from group J and group A, respectively. Pattern *i* lacks any intron in the basic region and leucine zipper region, and this pattern contains all genes of group C (10 genes), H (one gene), group I (two genes) and *VvbZIP15* in the group G. In summary, the intron position and splicing phase in the basic region and the leucine zipper region of grapevine bZIP gene family have been highly conserved during the course of evolution in each group, which is concordant with the early reports [2,8].

Furthermore, considering the patterns of *bZIP* gene family of rice, maize and grape, we find that although there are some differences of the splicing phase and the interrupted amino acids, the positions of the intron insert are highly conserved. This phenomenon suggested that the position of the intron insert in the conserved domain is important for the evolution and divergence of function in the gene family.

### Conserved structural features of VvbZIP transcription factors

**Figure 4:**
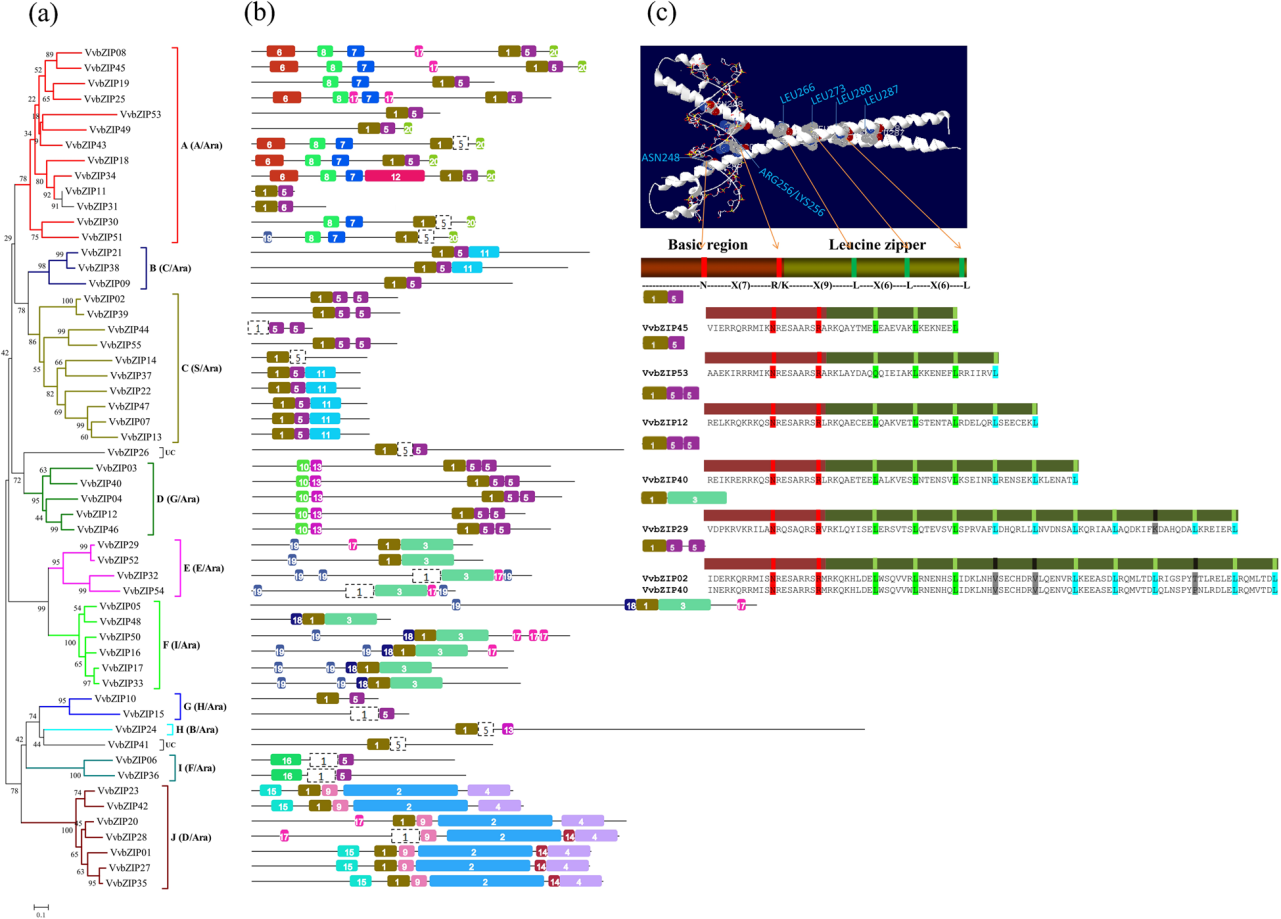
**Conserved motifs of VvbZIP proteins.** All conserved motifs of the VvbZIP proteins were identified by the MEME program. The protein structures **(b)** of VvbZIPs based on the presence of conserved motifs were arranged corresponding to the phylogenetic tree **(a)** of the VvbZIP proteins. Different motifs are highlighted with different colored boxes with numbers 1 to 20. Among them, the motif 1, 5, 9 and 13 mainly represented variations in the bZIP domain and the unusual motifs is shown in white. The sequence details of these conserved motifs are given in Additional file 3. The 3D structure and the variation of Leucine zipper region of VvbZIP proteins in length were described in **(c)**.

The bZIP domain is the core of the *bZIP* gene family members, which preferentially binds to the promoter of their downstream target genes on a specific cis-element (e.g. ABREs). However, the functional diversity of the bZIP gene family may also be contributed by additional domains in the bZIP proteins [46]. In total, 20 conserved motifs, including the bZIP domain, were identified in the VvbZIP proteins and their multilevel consensus amino acid sequences of motifs are listed in Additional file 3. The motif distribution corresponding to the phylogenetic tree of *VvbZIP* gene family is shown in Figure 4a,b. Motif 1 is the basic region and the hinge of the bZIP domain; whilst motif 5 (typically), 3 and 9 represent the variable motifs in the leucine zipper region across the bZIP gene family. For example, motif 3 only appeared in the group E and F; motif 9 appeared in the group J exclusively; and group C and D mostly harbored a double motif 5. Further, the number of Leucine units ranged from 3 to 11, and some of them were interrupted by one or two other units which are conserved with the same number of amino acids. Finally, nine gradients (I to IX) were defined (Additional file 4), and some of them showed group specificity, such as the gradient I which harbored the most members of group A, and gradient IX which harbored the most members of group C. Some typical cases and their corresponding motifs are shown in Figure 4c and may provide a greater understanding of the divergence and the roles of leucine zipper region in the bZIP gene family.

Beside the bZIP domain, each group of VvbZIP shares some motifs. For example, most members of the group A possess motifs 6, 7, 8 and 20; group B and C share motif 11; all members of the group D specifically harbor the motifs 10 and 13; group E shares motif 19 with group F which also possesses motif 18 exclusively. Motif 16 appeared in the group I, motifs 2, 4, 9, 14 and 15 are present in group J exclusively, whereas group G and H lack any additional motif.

Previous evidence suggested that most members of the group A of the bZIP gene family are involved in ABA-responsive signal-transduction pathway and abiotic stress response [4,25,47,48]. Typically, ABF/AREBs are master transcription factors that regulate ABRE-dependent ABA signaling involved in drought stress tolerance, but their activation requires posttranscriptional modification with ABA-dependent phosphorylation [25]. In the present study, two conserved sites, R/KxxS/T and S/TxxD/E, were verified as the key phosphorylation sites in VvbZIP proteins which are phosphorylated by a Ca^2+^ independent protein kinase and casein kinase II, respectively [5,32]. Furthermore, ABA-dependent multiple phosphorylation sites are also required for their activation. In our study (Figure 4b), motif 6, 7, 8 and 20 appeared in the group A VvbZIP exclusively and they widely represent the potential phosphorylation sites described above. Motif 6 harbors the R/KxxS/T and motif 7 harbors S/TxxD/E, presented as **R**Q[GNS]**S**, **T**[LF]D**E** and **R**[QE]P**T**, **T**LE[**DE**], respectively. Motif 8 and 20 contain just one of these sites, presented as **T**V[DE]**E** and **R**[TV][SL][**ST**], respectively. Moreover, these potential phosphorylation sites were also found in other groups of the VvbZIP gene family, presented as **R**QQ**T**/ **R**AL**S** in motif 4, **[RK]**[LV]Q**[SM]** in motif 11, **T**[DI][VAM][**DE**Q] in motif 15, and **R**[SA]S**S** in motif 19, respectively. In addition, other conserved motifs in bZIPs that may function as transcriptional activators, have also been found, including proline-rich, glutamine-rich, and acidic domains.

### Predicted DNA-binding site specificity of VvbZIP transcription factors

As one of the biggest transcription factor families in eukaryotes, bZIPs owe their DNA-binding ability and specificity to the bZIP domain. We aligned the amino acid sequences of each subfamily and compared them with those in Arabidopsis, rice and other important species in the plant kingdom, including *Physcomitrella patens* (moss), *Selaginella moellendorffii*, *Amborella trichopoda* (Additional file 5). The results show that some amino acid residues are highly conserved within each group across species in the tree of land plant life, indicating that those amino acid residues play crucial roles in the DNA-binding ability of the bZIP domain and have undergone purifying selection during their evolution. Experiments have shown that asparagine (numbered as -18), arginine (numbered as -10) and leucines (numbered as +1, +8, +15), were key and invariant sites of the bZIP domain, and their replacement causes new binding specificities [8,45,49]. In group F and in VvbZIP26 which seemingly belonged to group D in the phylogenetic tree (Figure 4a), the arginine site was replaced by Lys (just occurred in group F) and Ile, respectively, and is consistent with its subfamily members in other species (UC, Additional file 5). These replacements may have contributed to the emergence of a new binding specificity and independent course of evolution of this subfamily. In addition, some leucine sites were also replaced by other amino acids (for example, the third leucine site (+15) of most members in group C were replaced by Ile, and the second leucine site (+8) were mostly replaced by Met) which may have led to change in the binding specificity of these bZIP domains as well.

### Expression analysis of VvbZIP transcription factors at different developmental stages in specific organs in grapevine

**Figure 5:**
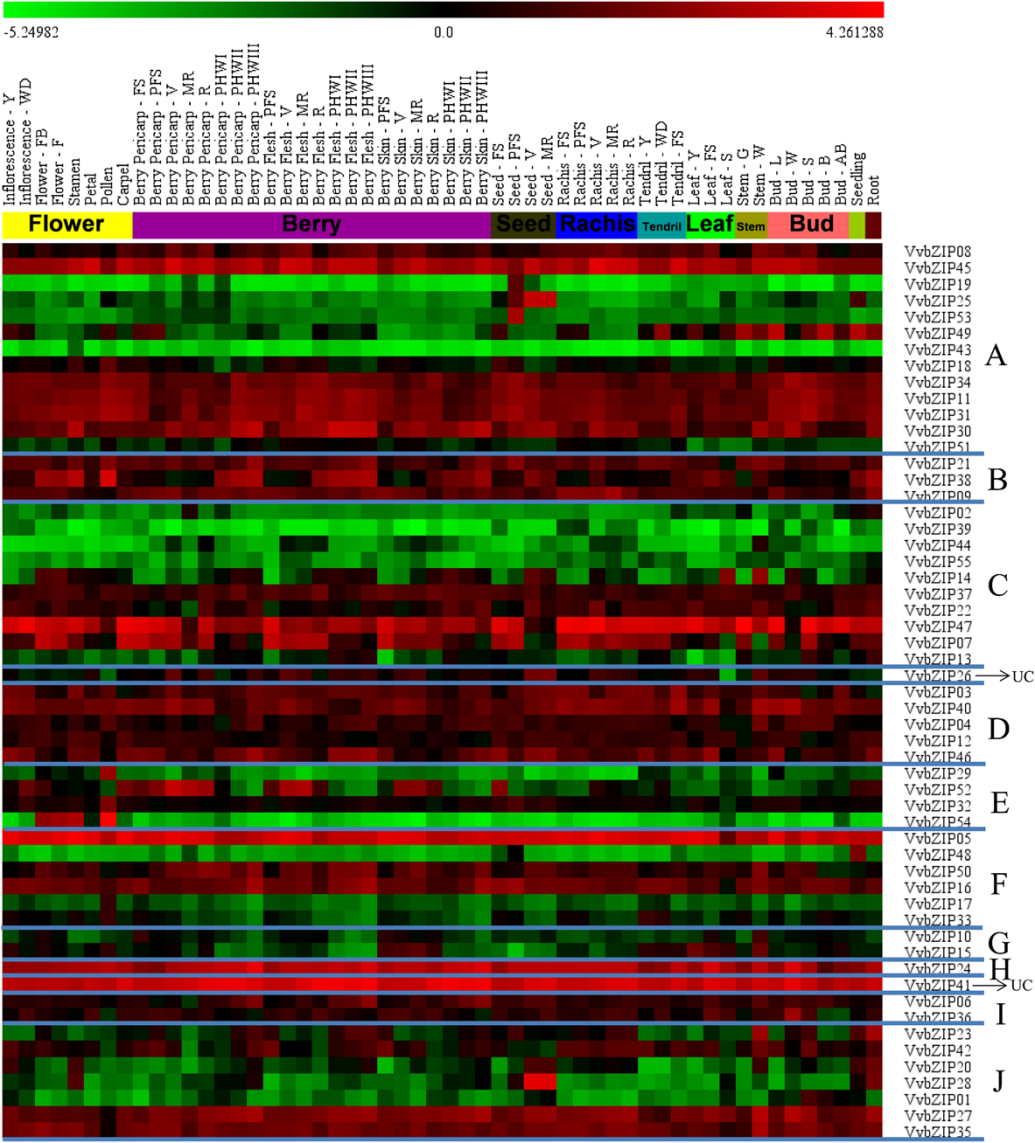
**Expression profiles of *VvbZIP *genes in 54 samples including green and woody tissues and organs at different developmental stages and some specialized tissues.** Fluorescence intensities values were log_2_-based. Genes were listed according to those in the phylogenetic tree and samples names are based on the gene expression atlas of grapevine [50].

Evidence has suggested that bZIP transcription factors are widely involved in the integration and development of many organs and tissues, such as seed maturation and germination [9], floral induction and development [11-18]. To obtain more insight into the temporal and spatial transcription patterns of grapevine *bZIP* gene family during grapevine development, a heatmap of the global transcription profile of the *VvbZIP* family was performed based on microarray data of the recent grape gene expression atlas on 54 samples (the sample information is described in Additional file 6) [50]. Fluorescence intensity values were log_2_-based to narrow the dynamic range of fluorescence intensities, thus giving a better appreciation of small differences in gene expression. All members of the grapevine *bZIP* gene family were surveyed. As illustrated in Figure 5, the *VvbZIP* gene family has a broad expression pattern across various developmental stages of grape organs and tissues. The *VvbZIP45*, *VvbZIP41*, *VvbZIP24* and *VvbZIP05* were abundantly expressed in all examined organs and tissues. In contrast, *VvbZIP39*, *43*, *55*, *51* and *44* were expressed at relatively low level. Moreover, *VvbZIP53*, and *VvbZIP19* were mainly expressed in seed at the stage of post-fruit set while *VvbZIP25* expression increased during the whole seed development. Notably, those three genes belonged to a subclade of the group A in the phylogenetic tree (Figure 1) harboring Arabidopsis homologous genes (*AtbZIP67*/*DPBF2*, *AtbZIP39*/*ABI5* and *AtbZIP15*), which have been confirmed to play an important role in ABA-mediated seed development, germination, and embryo maturation [51,52]. This suggests that *VvbZIP19*, *25* and *53* may have functions similar to their orthologous Arabidopsis genes during seed development in grapevine. Interestingly, the group E of *VvbZIP* showed peculiar expression in flower organs. For example, the *VvbZIP29* and *32* were mainly expressed in pollen and the *VvbZIP54* was highly expressed in Flower-FB, Flower-F, stamen and pollen, and the last member of group E, *VvbZIP52*, was highly expressed in the carpel but was relatively low in pollen, among others, indicating that the whole group E of grapevine bZIP gene family may be involved in flower development.

To survey the co-expression relationship between *VvbZIP* genes, Pearson’s correlation coefficients (PCC value) were calculated among bZIP genes based on the microarray data of 54 samples (Additional file 7), and showed that significant correlations existed between 213 pairs of *VvbZIP* genes at the 0.01 level (2-tailed), including 154 (72.3%) pairs showed significant positive correlation (PCC value ≧0.35) and 59 (27.7%) pairs showed significant negative correlation (PCC value ≦ - 0.35). Among them, just four pairs of genes, *VvbZIP19* (Group A)/*53* (Group A), *VvbZIP25* (Group A)/*28* (Group J), *VvbZIP11* (Group A)/*31* (Group A) and *VvbZIP29* (Group E)/*54* (Group E), showed strong positive correlations with PCC value of > 0.9, and seemed to belong to the same group, indicating that they probably contribute to some redundant function or are involved in the same regulation network of biological process.

In addition, we carefully examined the differentially expressed *VvbZIP* genes in the development of various organs (Additional file 8), suggesting that the *VvbZIP* genes are involved in seed development (e.g., *VvbZIP25*, *14*, *20*, *53 19*, *28*, *7*, *47* and *52*), leaf and stem development (e.g., *VvbZIP20*, *23*, *44*, *14* and *17*), berry maturity (e.g., *VvbZIP2*, *44*, *42* and *49*) and bud dormancy. Interestingly, *VvbZIP 14*, *44*, *20* and *47* were involved in the development of more than one organ, indicating that they may participate in some basic biological processes.

### Analysis of *VvbZIP* gene expressions by quantitative real time PCR

**Figure 6:**
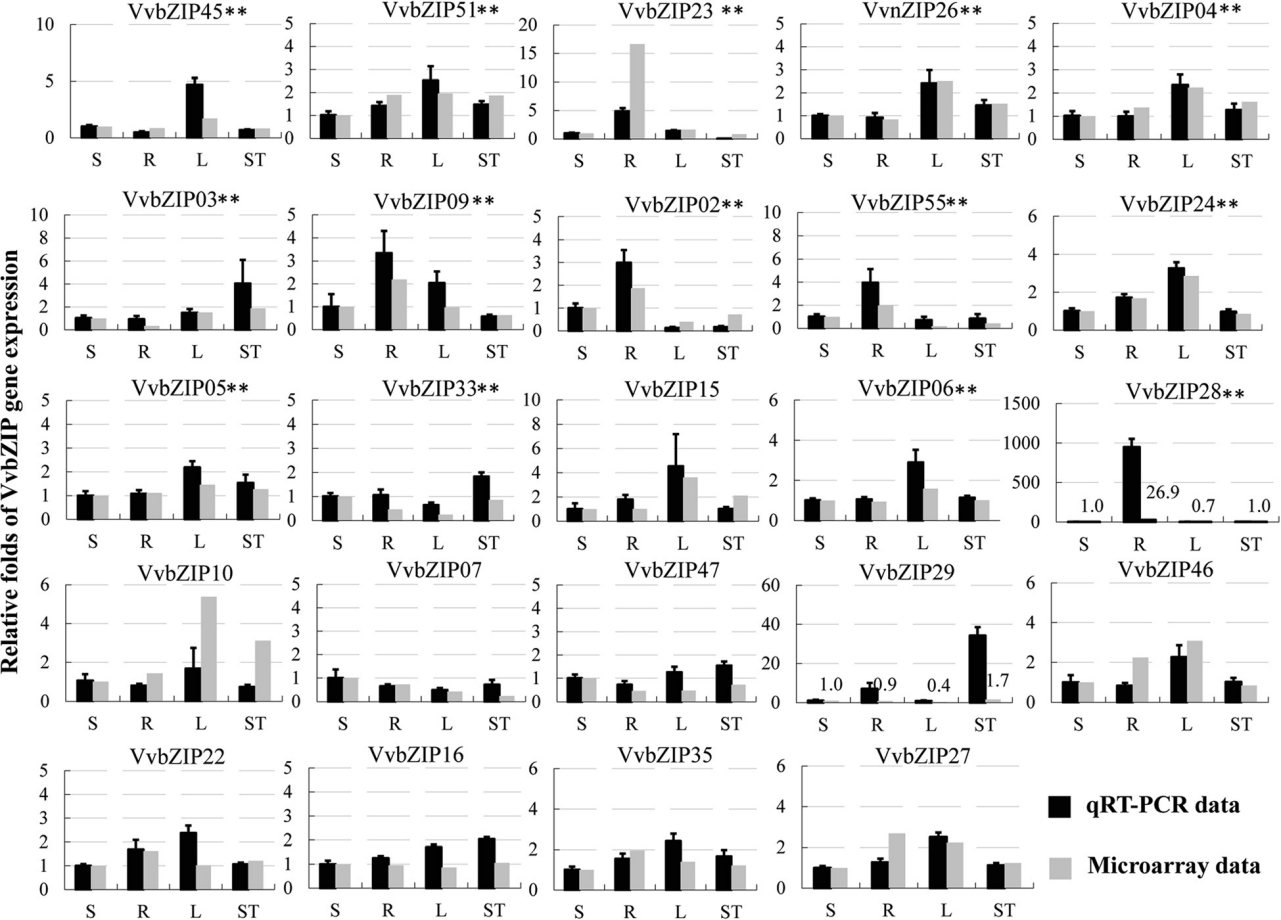
**The comparison between quantitative real-time RT-PCR data and Microarray data.** The relative expressions of 24 selected *VvbZIP* genes were performed with quantitative real-time RT-PCR (qRT-PCR). The relative expression of stem/green stem was set up as 1. The y-axis indicates the folds of gene expression relative to stem. The expression in significant agreement between qRT-PCR data and Microarray data were marked with stars based on PCC value in 0.01 (two stars). Characters on the x-axis indicate various tissues, S, stem/green stem; R, root/root; L, leaf/ mature leaf; ST, shoot tip/ bud after-burst. The black bars denote the qRT-PCR data, and the gray bar denote the microarray data.

Quantitative real-time PCR (qRT-PCR) was performed on four different organs (stem, root, leaf and shoot tip) of Pinot Noir PN40024 grapevine plants for VvbZIP genes, and the results obtained were compared with microarray data of the cultivar ‘Corvina’ [50] with the same organs at the corresponding phonological phases (green stem (Stem-G), root (Root), mature leaf (Leaf-FS) and bud after-burst (Bud-AB)) (Figure 6). The results show that the expression profiles of *VvbZIP* genes from qRT-PCR were mostly in agreement with the microarray data based on calculation of PCC value. This means that similar spatiotemporal expression of members of the *VvbZIP* gene family is generally conserved among different cultivars like Corvina and Pinot Noir, grown in very different conditions, i.e. in the field and in vitro, respectively. Our results provide evidence for selecting candidate genes for further characterization in tissue/organ specificity.

### Expression of *VvbZIP* genes under drought and heat stress

**Figure 7:**
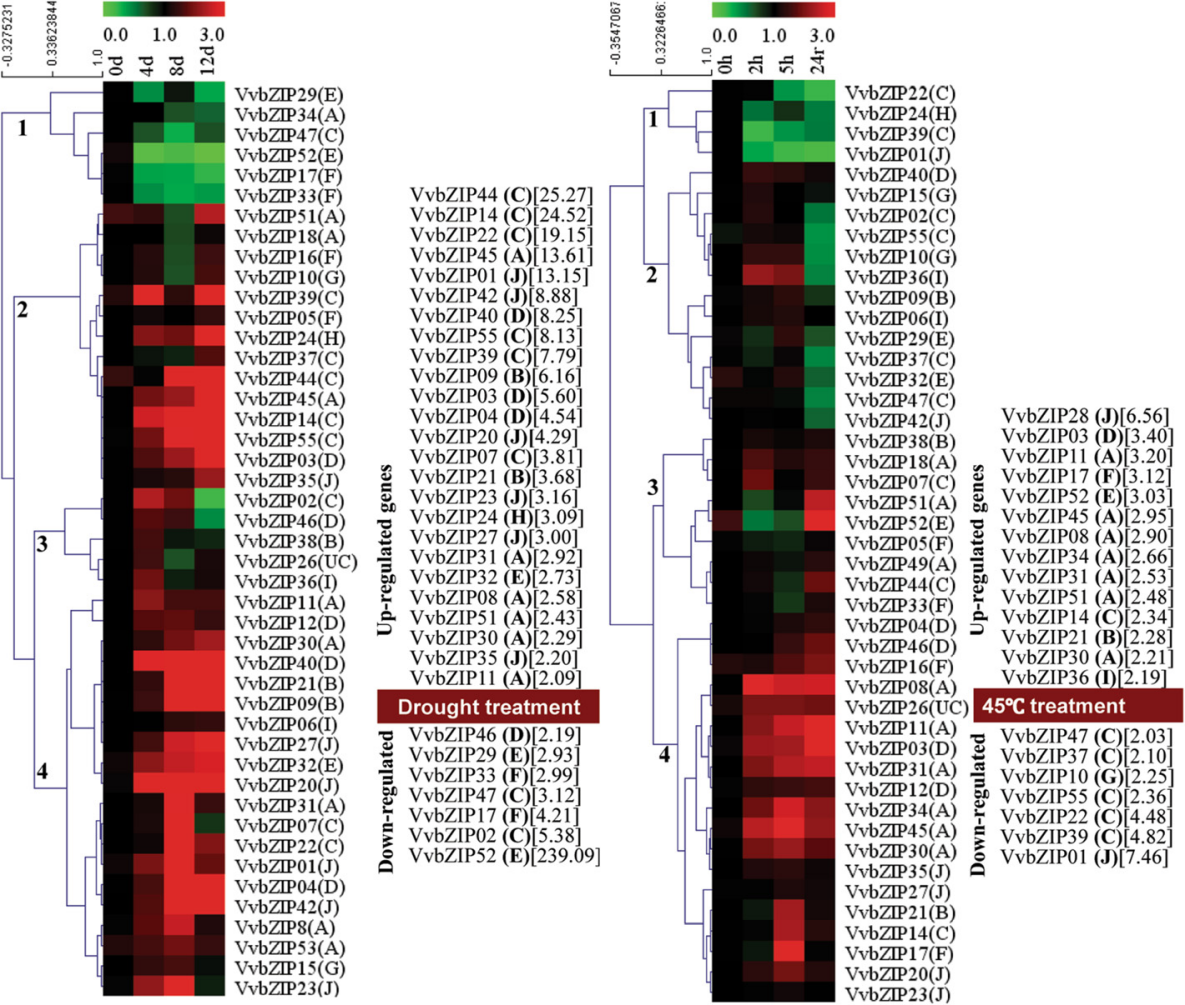
**Quantitative RT-PCR analysis of the VvbZIP gene expression in grapevine leaves (sequenced genotype, PN40024) in response to drought stress and 45°C heat stress.** The normalized relative expression level of the 0 d treatment timepoint was set up as 1. The y-axis indicates the relative folds of gene expression compared with the control. Leaves collected at 0, 4, 8 and 12 d post-drought or at 0, 2, 5 h post heat stress and 24 h recovery. Means are calculated from technical triplicate qRT-PCR measurements within three biological replicates.

Plants and crops are frequently challenged by abiotic stress, such as drought stress and high temperature. Some evidence has suggested that bZIP proteins are widely involved in signaling and responses to abiotic/biotic stimuli [2,26,30,53], but limited information was available on bZIP involvement on stress responses in grapevine. In the present study, long-term drought condition (0, 4, 8 and 12-day) and short-term heat stress (0, 2, 5 hours and 24-hour recovery) on grapevine seedling treatment had marked effect on the expression profile of 47 out of 55 (85.5%) *VvbZIP* genes for which we were able to design primers using for qRT-PCR analysis (Figure 7). As illustrated in Figure 7, both heatmaps (responses to drought and heat) could be divided into four clusters. For the drought treatment, cluster 1 contains 6 (13.3%) members of detectable *VvbZIP* genes, which were widely down-regulated by drought treatment. Cluster 2 contains genes highly induced at 4 days after drought treatment, but down-regulated at 8 days and 12 days after drought treatment. Cluster 3 mainly consists of genes with the highest expression level at 8 days after drought treatment. Cluster 4 has 15 (33.3%) members which were mainly up-regulated with the increased degree of the drought treatment, and particularly, *VvbZIP44*, *45*, *14*, *55* and *03* were highly induced at 12 days after drought treatment. Overall, 25 *VvbZIP* genes were up-regulated at least two fold after drought treatment relative to the control and ranged from 2 to 25 fold, 7 *VvbZIP* genes were down-regulated at least two fold after drought treatment relative to the control and ranged from 2 to 239 fold.

For the heat stress treatment and recovery, cluster 1 has three (6.7%) members of 45 detectable *VvbZIP* genes, which were widely down-regulated after 45°C treatment and recovery. The members of cluster 2 were mainly down-regulated after 24 hour of recovery. The members of cluster 3 were mainly down-regulated after 45°C treatment for 2 or 5 hours, but were up-regulated after recovery for 24 hours. Cluster 4 expressed highest across all points of heat stress treatment. Overall, 14 *VvbZIP* genes were up-regulated at least two folds after 45°C treatment or recovery, the fold change ranged from 2 to 7; 7 *VvbZIP* genes were down-regulated at least two fold and ranged from 2 to 7.

Interestingly, some up-regulated genes typically induced by drought were down-regulated during the heat stress or in the recovery process, such as *VvbZIP01*, *39*, *22* and *55*. On the contrary, some genes down-regulated by the drought treatment were up-regulated by the heat stress or recovery, such as *VvbZIP52* and *17* (Figure 7). This result suggests that different *VvbZIP* genes are involved in these two different biological pathways in response to drought and heat stress.

Furthermore, the Pearson’s correlation coefficient (PCC value) among pairs of *VvbZIP* genes based on all of the qRT-PCR expression data in the drought and heat stress conditions (Additional file 9) showed that 66 pairs of genes showed significant correlation at the 0.01 level (2-tailed), including 43 (65.2%) pairs showed significant positive correlation (PCC value ≧0.99) and 23 (34.8%) pairs showed significant negative correlation (PCC value ≦ - 0.99). the fact that 17 positive and 13 negative pairs of correlations between drought and heat stress conditionsuggests that some *VvbZIP* genes may also be involved in some common pathways in responses to drought and heat stresses.

## Discussion

Increasing evidence suggests that the bZIP proteins play crucial roles in various developmental and physiological processes in plants, such as seed maturation [9,10], flower development [11-18], stress signaling [25,31,32] and pathogen defense [30]. Much of the research into the functions of the bZIP genes have been characterized in model plants, especially in Arabidopsis, and some crops including rice, barley and Sorghum [2,4,7,54,55]. However, very little is known about this family in other species.

In this report, we exhaustively identified the bZIP genes in grapevine genome, and examined their evolutionary history and high-quality expression profiles across the different developmental stages of various kinds of tissues and under drought and heat stress conditions. The information provides a comprehensive overview of the bZIP gene family in grapevine and lays the foundation for future functional characterization of individual VvbZIP genes.

### The distribution and colinearity of grapevine bZIP gene family reflected evolutionary imprint of grapevine genome

Grapevine is evolutionarily the earliest-diverging lineage of rosids and is a sister clade to eurosids I and II [36,42]. The species is believed to have evolved about 60 MYA, and its genome sequence revealed that it has not undergone any whole genome duplication since the gamma genome duplication. Therefore, it serves as a good resource for understanding the evolution and functional divergence of genes in rosids. The bZIP transcription factor family has evolved with a long history, which can be at least traced back to the earliest land plants, such as moss (*Physcomitrella patens*), which contains at least 37 bZIPs [55]. The grapevine, described as a ‘palaeo-hexaploid’, has homologous triplets in their genome and is believed to have resulted from a genome fusion of a tetraploid and a diploid genome and subsequent diploidization [35]; At the gene family level, the phylogenetic analysis of nucleotide binding site (NBS) gene family members [36], supported the fusion hypothesis, and suggested that the ancestral hexaploid genome of grapevine was derived from an allopolyploidization of two ancestral genomes: Va and Vc. However, the analysis of the calcium-dependent protein kinases (CDPK) gene family showed no indication of such imprint on fusion and divergence [56], and it may be because the gene family (17 members) is not large enough in tracing the evolutionary history, or the retention and loss after fusion was unequal. In this study with a large bZIP gene family (55 members), the distribution and gene duplication events of *VvbZIPs* (Figure 2), are very un-uniform. Five chromosomes (9, 10, 11, 16 and 17) harbor no bZIP gene, and chromosome 15 harbored just one. Interestingly, chromosomes that lack bZIP genes seem to belong to the Vc genome, and this indicates that the grapevine bZIP gene family suffered extensive gene loss after fusion and this loss mainly occurred in Vc genome. The 12 homologous pairs of *VvbZIP* genes plus two triplets (*VvbZIP07*/*13*/*47* and *VvbZIP14*/*22*/*37*) occurred within the same homologous blocks and this partially supports the fusion hypothesis in the grapevine genome [35,36].

Furthermore, Comparing the number of *VvbZIP* genes with that of other species, it is ~26% less than that in Arabidopsis, and ~38% less than that in rice. This is probably because grapevine did not undergo any whole genome duplication (WGD) events after the gamma WGD as shown in Arabidopsis, or the WGD after split between eudicots and monocots which happened in rice [57].

### The colinearity-orthologues of the bZIP genes between grapevine and Arabidopsis

Comparative genomic analyses across different taxa allows the transfer of functional information from a well-characterized taxon, such as the model plant Arabidopsis, to another less-studied taxon, like grapevine. This is true with bZIP genes, many of which have been well-characterized in Arabidopsis, but few of which have been studied in grapevine. Therefore, the determining orthologous relationships between the Arabidopsis bZIP genes with those in grapevine (Table 1) will allow the grapevine researchers to make a “best guess” of their functions. Most orthologues clustered at the same clade in the phylogenetic tree (Figure 1) share common conserved motifs (data not shown). In Arabidopsis, *AtbZIP36* (*AREB1*), *38* (*AREB2*), and *37* (*ABF3*) have been shown to be master transcription factors that cooperatively regulate ABRE-dependent ABA signaling involved in drought stress response [25]. In grapevine, their orthologues are *VvbZIP8* and *VvbZIP45*, which were also shown to be highly inducible in response to drought stress in this study (Figure 7). Moreover, Nicolas et al. [39] recently demonstrated that *VvbZIP45* transcripts mainly accumulated in the berry during ripening, and were up-regulated by ABA [39]. Comparative genomic analyses between grapevine and Arabidopsis provides a good reference for studying the biological functions of VvbZIPs.

### The grapevine bZIP gene family may participate in different aspect of organ development

The in-depth analysis on a high-quality microarray data [50] (Figure 5) indicated that bZIP proteins may widely participated in the differentiation and development of many organs and tissues, and this was verified using qRT-PCR (Figure 6). Tissue-specific expression patterns include *VvbZIP19*, *25* and *53* in seed development, *VvbZIP29*, *32* and *54* in flower development, indicating their primary roles in the morphogenesis and organ development. These results may lead to more directed understanding the function of these *VvbZIP* genes in grapevine development biology.

### Diverse *VvbZIP* genes are involved in responses to drought and heat stress

Previous studies have shown that bZIP genes play an important role in plants in response to various stresses, such as drought, salt, freezing temperature, and heat [2,4,46]. In this study, the expression profiles of *VvbZIP* genes under drought and heat stresses (Figure 7) revealed that grapevine bZIP genes are widely involved in responding to the drought and heat stress. Interestingly, some drought up-regulated genes were down-regulated in heat stress, which indicates that two sets of *VvbZIP* genes are involved in response to the drought and heat stress, respectively, others are co-expressed in response to both heat and drought stresses, and those genes may participate in shared roles between drought and heat stress. The data of the Pearson’s correlation coefficient (PCC value) among VvbZIP gene pairs also supports this notion (Additional file 9).

## Conclusions

Grapevine (*Vitis vinifera*) genome contains 55 members in the *bZIP* gene family, and these genes are located in 14 chromosomes. Genome distribution, gene organizations and gene structures suggested a complex evolutionary history of this family in grapevine. The grapevine bZIP family is greatly contributed by the segment/chromosomal duplications, which may be associated with the grapevine genome fusion events, their expression profiles in various tissue/organ and their development stages and responses to drought and heat stress condition demonstrated that this gene family is widely involved in grapevine organ development and drought- and heat-responses. The overall picture of this gene family and their potential involvement in growth, development and stress responses will facilitate further research on the bZIP gene family regarding their evolutionary history and biological functions.

## Methods

### Genome-wide identification of bZIP transcription factors in grapevine

To identify all bZIP transcription factor genes in grapevine (*Vitis vinifera*), all annotated proteins were downloaded from the latest database version (V1) of the 12X assembly of grapevine genome (http://genomes.cribi.unipd.it/). The Hidden Markov Model (HMM) profile of the bZIP domain (PF00170) downloaded from Protein family (Pfam) (http://pfam.sanger.ac.uk/) was used for identification of the bZIP genes from the downloaded database of grapevine genome using HMMER3.0 [58]. All output genes with default (< 1.0) E-value were collected and the online software SMART (http://smart.embl-heidelberg.de/smart/set_mode.cgi?GENOMIC=1) was used to confirm the integrity of the bZIP domain with E-value < 0.1 [59] and the incorrectly predicted genes were rejected. Finally, the non-redundant and confident genes were gathered and assigned as grapevine bZIP genes. Furthermore, the same methods were also used for detecting of possible additional bZIP signatures in other database, such as Genoscope and NCBI, or identification of poplar bZIP genes. The protein sequences of grapevine, poplar and Arabidopsis bZIP transcription factors were deposited at Additional file 10.

### Phylogenetic analysis and identification of conserved motifs of the VvbZIP family

The conserved bZIP domain sequences of were extracted from bZIP proteins using online program SMART (http://smart.embl-heidelberg.de/) for phylogenetic analysis. Multiple alignments of protein sequences were executed by Clustal X 1.83 program [60]. Phylogenetic trees were constructed using MEGA 5.0 by the Neighbor-Joining (NJ), Minimal Evolution (ME) methods and the bootstrap test carried out with 1000 iterations, but only NJ tree was presented because all trees are consistent. The MEME program (version 4.8.1, http://meme.nbcr.net/meme/cgi-bin/meme.cgi) was used for identification of additional conserved motifs outside the bZIP domain, with the following parameters: number of repetitions: any; maximum number of motifs: 50; and the optimum motif widths: 6–200 amino acid residues.

### Genomic structures, chromosomal locations and gene duplications for *VvbZIP* genes

Each of the *VvbZIP* gene was positioned on grape chromosome derived from the CRIBI (http://genomes.cribi.unipd.it/) using the programmed Perl script. The homeologous chromosome segments resulting from whole-genome duplication or fusion events were shown in the same color according to Jaillon et al. [35]. Gene structure display server program (GSDS, http://gsds.cbi.pku.edu.cn/index.php) was exploited to illustrate exon/intron organization for the bZIP genes by aligning the cDNAs with their corresponding genomic DNA sequences [61]. For detecting the gene duplication events, the MCScanX software (http://chibba.pgml.uga.edu/mcscan2/) was used and the E-value was set under 1E-5. The other manipulations followed the operation manual.

### Plant materials preparation and treatment

In vitro grapevine plants (*V. vinifera*, the sequenced genotype PN40024) were maintained in vitro on ^1^/_2_ MS medium supplied with 0.3 mg/L indole 3-butyric acid under a 16/8 h photoperiod (100 μmol m^-2^ s^-1^) at 25°C in the culture room.

For drought stress treatment, one-month-old tissue cultured PN40024 plants were acclimatized in the lighted growth chambers [16-h light (25°C)/8-h dark (23°C), relative humidity of 60–70%, and light intensity of 20 μmol m-2 s-1 for 15 days, then watered regularly for 10 days, and this was set as the 0 d of treatment. One group of plants was then kept watered as the control, the other three groups were stopped being watered (the drought treatment) for a duration of 4, 8 and 12 days, respectively, and every duration of treatment had a corresponding regularly-watered control. For each treatment, three independent plants (biological replications) were sampled.

For heat stress treatment, in vitro-grown one-month old plants were directly placed in the 45°C lighted growth chamber (light intensity of 20 μmol m-2 s-1), excepting the temperature, the other conditions were the same as in the drought treatment. After heat treatment for 0, 2, 5 hours, plants were kept for 24 hours at 25°C for rapid recovery [27]. Each treatment contained three treated plants, or biological replicates, and three corresponding non-treated control.

For tissue specificity expression of *VvbZIP* genes, tissues of root, stem, leaf (4-6^th^ leaves from the tip) and shoot tips were harvested separately from three one-month-old tissue in vitro-grown plants of PN40024 (three biological replicates). After freezing in liquid nitrogen, all plant materials were stored at -80°C.

### RNA extraction, primer design and quantitative real-time PCR

Total RNA was extracted using Huayueyang Quick RNA isolation Kit (Cat.No.: ZH120; Huayueyang biotechnology, Beijing, China), all protocols followed the manufacturer’s procedure. To make sure that there have no DNA contamination, the traditional digestion protocol was used to eliminate DNA in the sample (rDNase®, Cat.No.: D2270A, TaKaRa Biotechnology, Dalian, China). The qualities and quantities of RNA were determined by agarose gel electrophoresis and Nanodrop ND-1000 Spectrophotometer (Thermo Fisher Scientific Inc.; USA). For cDNA synthesis, 500 ng high quality total RNA was reverse transcribed with oligdT and random primers using SuperScriptIII Reverse Transcriptase (TaKaRa, Dalian, China) according to the manufacturer’s instructions.

Gene-specific primers for the *VvbZIP* genes were designed according to the predicted mRNA sequence using Beacon Designer 7.0 software (Premier Biosoft International, USA). To enhance specificity of the primer, most of them were cross-blasted the ORF and 3’UTR of *VvbZIP* genes, all of the primers were tested with PCR amplification, electrophoresis gels, melting curve analysis and the products of PCR amplification were sequenced at least three times, the maximum deviation of which were less than 3 base pairs. The sequences of primers and their products were listed in Additional file 11, and noted that primers for *VvbZIP28*, *49*, *52* and *53* were not very specific, so it was just used in one or two treatment for determining the gene expression.

For quantitative real-time PCR (qRT-PCR) assay, cDNA was diluted to about 100 ng/μl with ddH_2_O. The qRT-PCR was carried out on ABI 7300 Real-time PCR System (Applied Biosystems) using SYBR® Premix Ex TaqTM (TaKaRa Code: DRR420A, TaKaRa, Dalian, China). The reaction system (total volume of 20 μl) was contained: 10 μl SYBR® Premix Ex TaqTM (2×), 0.4 μl of each primer (10 μM), 0.4 μl ROX Reference Dye (50×), 1 μl of template (about 100 ng/ μl), finally, add the ddH_2_O to total volume 20 μl. The program were performed as the following steps: 95°C/30 s for pre-denaturation (step 1), 95°C/5 s for denaturation (step 2), 60°C/34 s for primer annealing/extension and gathering the fluorescent signal (step 3), then go to step 2 for 40 circles, each reaction was completed in triplicate for the accuracy of results. At the end, the melting curve analysis was executed for verifying the specificity of the primer with the following program: 95°C/15 s, 60°C/1 min, 95°C/15 s. Transcript levels were normalized against the average expression of *VvACTIN* gene (VIT_12s0178g00200), and the baseline and threshold cycles (Ct) were automatically determined by the software of the system. For the data analyses, 2^-ΔΔC^ method [ΔΔCt = (Ct_target gene_ – Ct_Actin gene_)_treatment_- (Ct_target gene_ – Ct_Actin gene_)_control_] was used for calculating the relative expression of *VvbZIP* genes. To visualizing the relative fold difference, all data were normalized based on setting up the relative expression level, the expression level of 0-point treatments for drought stress and heat stress and stem for plant tissue specificity was set as 1, “above 1” and “below 1” are considered as upper, or down, regulation in drought and heat treatment, and more, or less abundant in tissue specificity assay, respectively. The data (0 to 3 folds) were displayed and clustered by MeV v4.8 software (http://www.tm4.org/), and the genes induced by each stress with their maximum folds (> 2 folds) of relative expression level were accounted and performed in the Figure 7.

### Microarray data analysis

To understand the spatial and temporal expression patterns of *VvbZIP* genes during development, a high-throughput microarray data, from recent research [50], was employed for further analysis (Additional file 12). In the data sets, 54 samples, including green and woody tissues and organs at different developmental stages as well as specialized tissues such as pollen and senescent leaves was determined (Additional file 6). The heatmaps were made by MeV v4.8 software (http://www.tm4.org/) [62].

### Statistics analysis

To determine the co-expression relationship of *VvbZIP* genes in various tissues/organs development stages and drought and heat responses, the Pearson’s correlation coefficient (PCC) values among *VvbZIP* gene pairs were calculated based on the microarray data of various tissues/organs development (Additional file 7) and qRT-PCR data of drought and heat stress (Additional file 9) using program Statistical Product and Service Solutions (SPSS v20.0). The significant correlation at the 0.01 level (2-tailed) was determined and denoted with red (significant positive correlation) and blue (significant negative correlation) background.

## Abbreviations

ABREs: ABA response elements; WGD: Whole genome duplication; MEME: Multiple expectation maximization for motif elicitation; qRT-PCR: Quantitative reverse transcription polymerase chain reaction.

## Competing interests

The authors declare that they have no competing interests.

## Authors’ contributions

JYL performed all the bioinformatics analysis and experimental work, and drafted the manuscript; NNC helped in experimental works and data analysis; CF and BC helped in bioinformatics analysis, data mining and management; SDS, GBT and MP shared and analyzed the microarray data and contributed to revision of the manuscript; ZMC conceived the project, provided overall supervision of the study and drafting the manuscript. All authors read and approved the final manuscript.

## Supplementary Material

Additional file 1 **The gene structure of VvbZIPs. (a)** The intron-exon arrangement of *VvbZIP* genes; **(b)** the number of exons in *VvbZIP* genes. Click here for file

Additional flie 2 The position and pattern of introns within bZIP domain. Click here for file

Additional file 3 Multilevel Consensus Sequence and their logo of VvbZIP proteins as predicted by MEME program. Click here for file

Additional file 4 The length of Leucine zipper region in bZIP domain of VvbZIP proteins. Click here for file

Additional file 5 Alignment of bZIP domain of VvbZIP proteins. Click here for file

Additional file 6 **Description of organs and tissues of *Vitis vinifera *** **cultivar Corvina, from a vineyard in the Verona province (Montorio).** Click here for file

Additional file 7 **The Pearson correlation coefficient (PCC value) between *VvbZIP *** **genes based on the microarray data of various tissues/organs development.** Click here for file

Additional file 8 **Differentially expressed genes of grapevine bZIPs in various kinds of tissues.** The microarray data was normalized based on setting up the expression level of first development stage of each tissue as 1, the folds of differential expression was subsequently calculated. The genes above and below the colored tissue bar indicated the up-regulated and down-regulated genes during this tissue development, respectively. The expression fold was presented at bracket along with each *VvbZIP* gene. The genes fluctuated more than 5 folds was highlight with red characters. Click here for file

Additional file 9 **The Pearson correlation coefficient (PCC value) between *VvbZIP *** **genes based on the qRT-PCR data of drought and heat (45°C) treatment.** Click here for file

Additional file 10 Primer sequences used for real-time PCR analysis. Click here for file

Additional file 11 Protein sequences of grapevine, poplar and Arabidopsis bZIP transcription factors. Click here for file

Additional file 12 **High-throughput microarray data of *VvbZIP *** **gene expression.** Click here for file
