# Supplementary material for: Genome-wide analysis and expression profile of the bZIP transcription factor gene family in grapevine (Vitis vinifera)
Source: BMC Genomics. 2014 Apr 13;15:281. doi: 10.1186/1471-2164-15-281 (PMC4023599; doi:10.1186/1471-2164-15-281)

Additional file 1. The gene structure of VvbZIPs. (a), The intron-exon arrangement of VvbZIP genes; (b), The number of exons in VvbZIP genes.

(a)

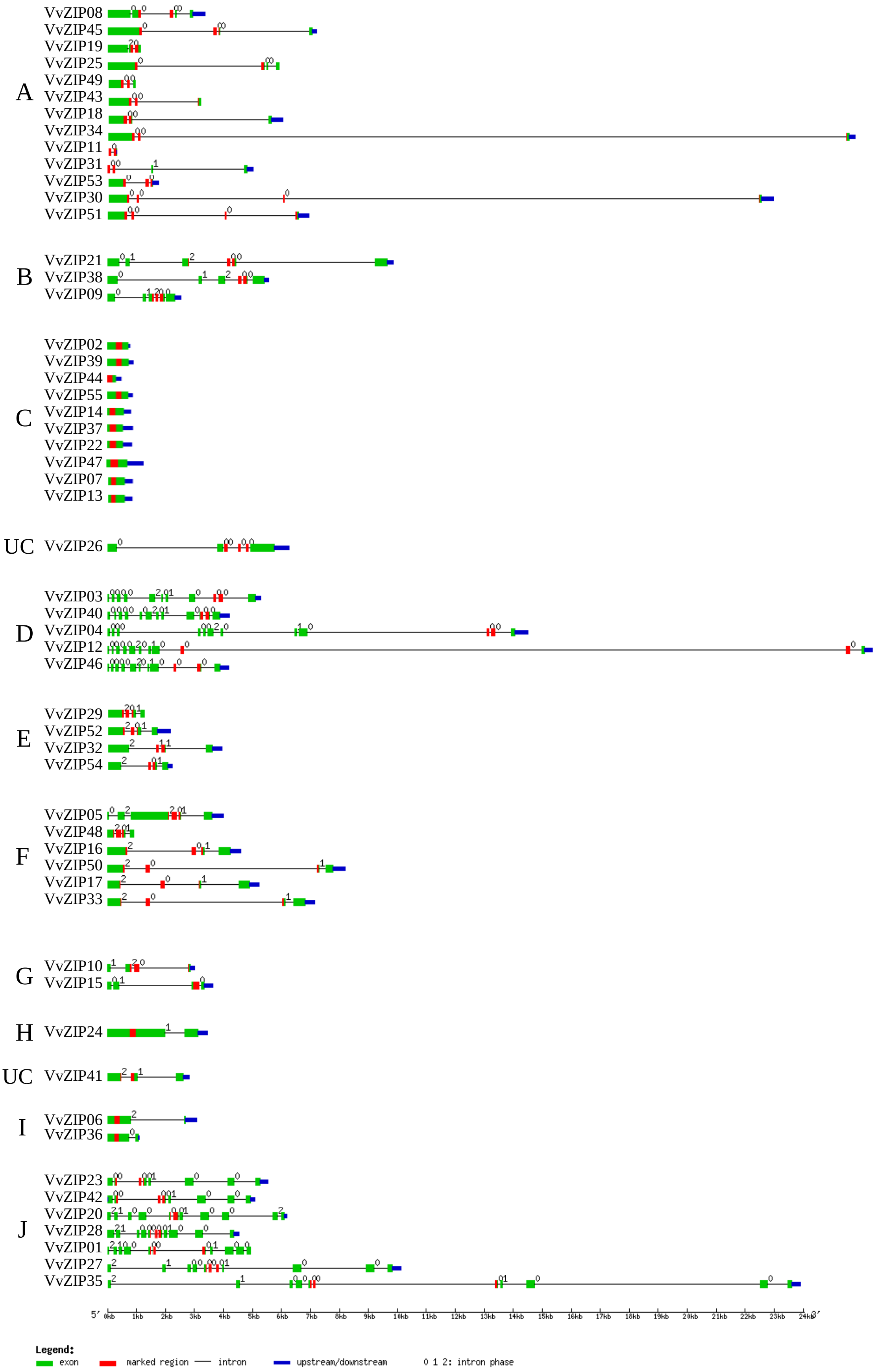

(b)

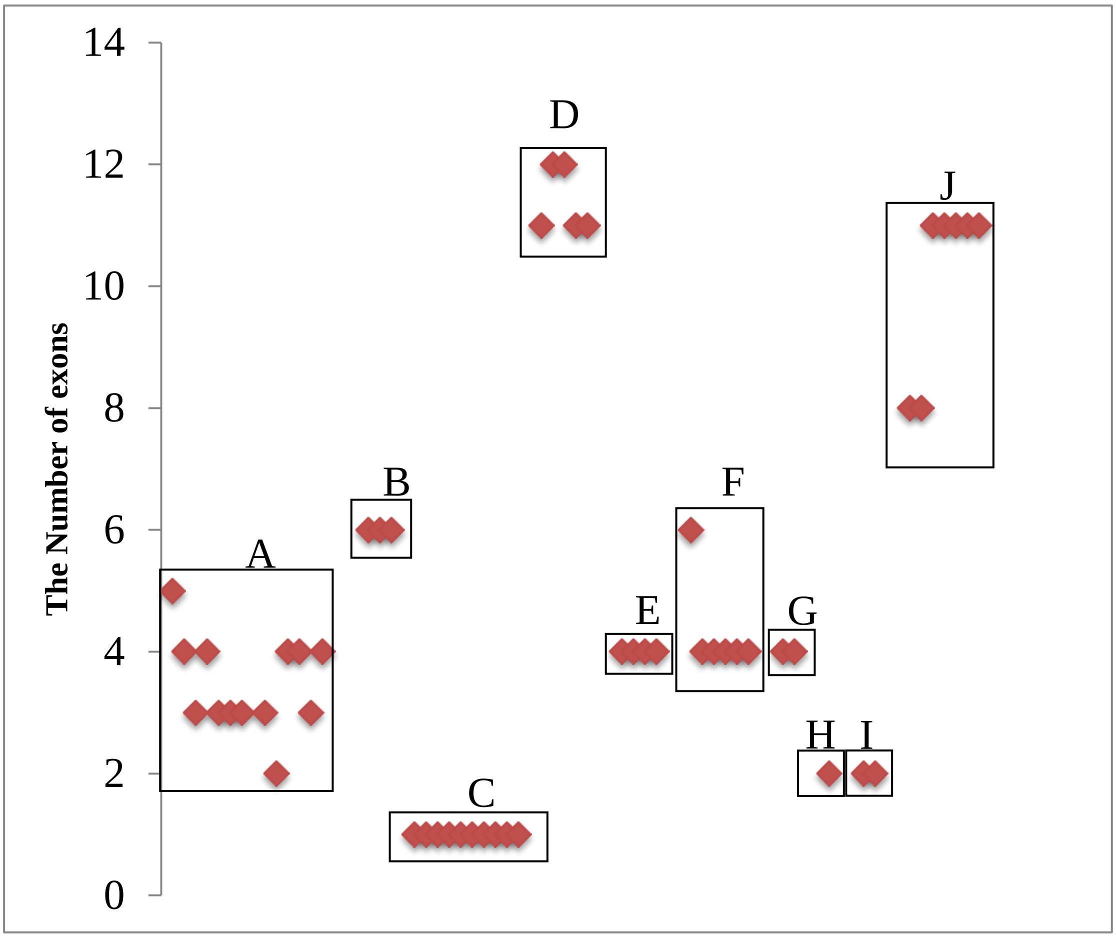

Supplement: Additional file 1 — The gene structure of VvbZIPs. (a) The intron-exon arrangement of VvbZIP genes; (b) the number of exons in VvbZIP genes. [file 1471-2164-15-281-S1.pdf]
