# Supplementary material for: Genome-wide analysis and expression profile of the bZIP transcription factor gene family in grapevine (Vitis vinifera)
Source: BMC Genomics. 2014 Apr 13;15:281. doi: 10.1186/1471-2164-15-281 (PMC4023599; doi:10.1186/1471-2164-15-281)

**Additional file 2. The position and pattern of introns within bZIP domain**

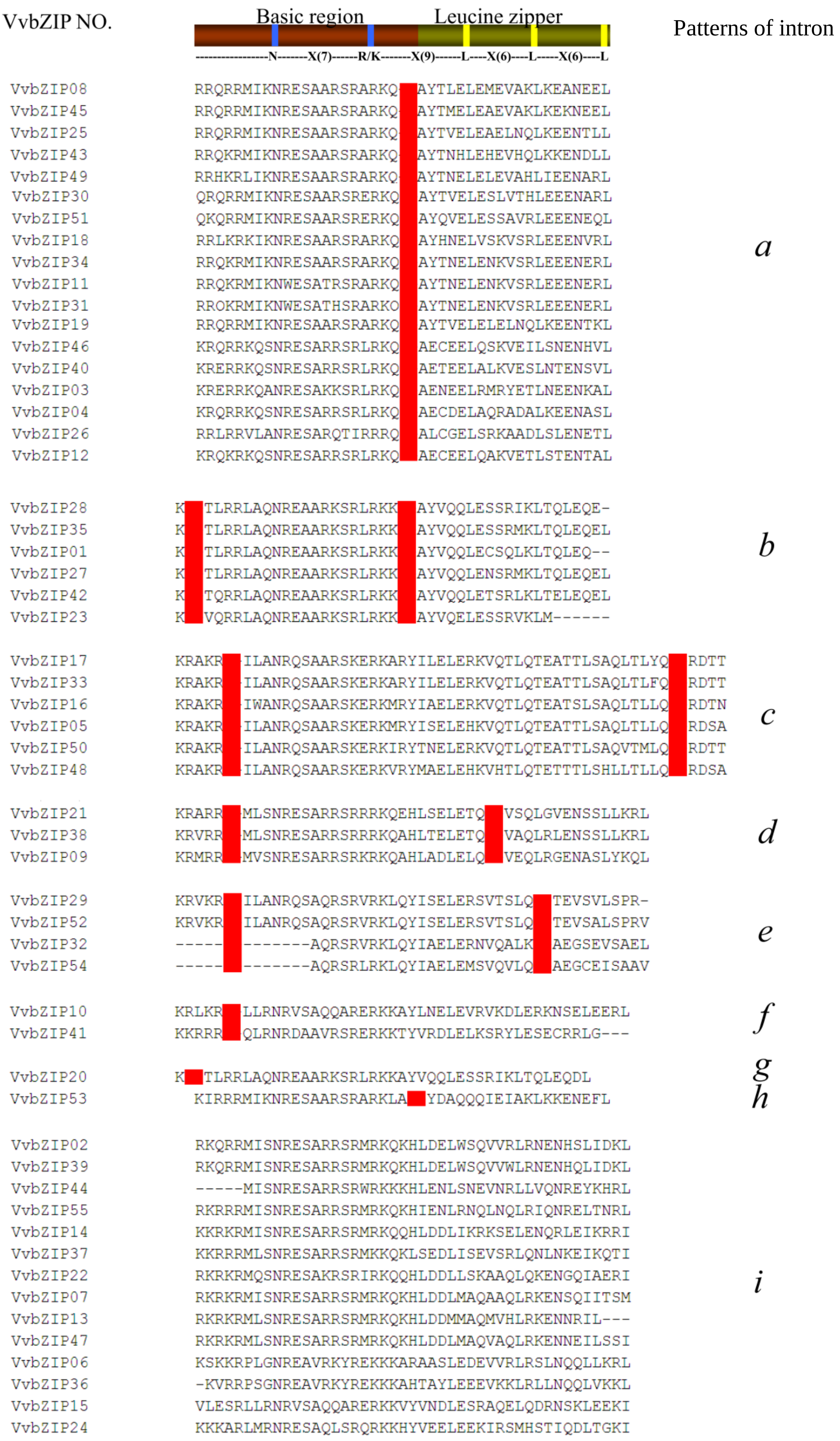

Supplement: Additional flie 2 — The position and pattern of introns within bZIP domain. [file 1471-2164-15-281-S2.pdf]
