# Supplementary material for: Genome-wide analysis and expression profile of the bZIP transcription factor gene family in grapevine (Vitis vinifera)
Source: BMC Genomics. 2014 Apr 13;15:281. doi: 10.1186/1471-2164-15-281 (PMC4023599; doi:10.1186/1471-2164-15-281)
Supplement: Additional file 3 — Multilevel Consensus Sequence and their logo of VvbZIP proteins as predicted by MEME program. [file 1471-2164-15-281-S3.docx]

**Additional file 3.** Multilevel Consensus Sequence and their logo of VvbZIP proteins as predicted by MEME program.

| Motif No. | E-value | Multilevel Consensus Sequence and their logo |
| --- | --- | --- |
| **Motif 1** | **1.6e-753** | DE[KR][RK]Q[RK]RM[IL][SKA]NRESA[RA]RSR[ALE]RKQA[YH][TL]NELE  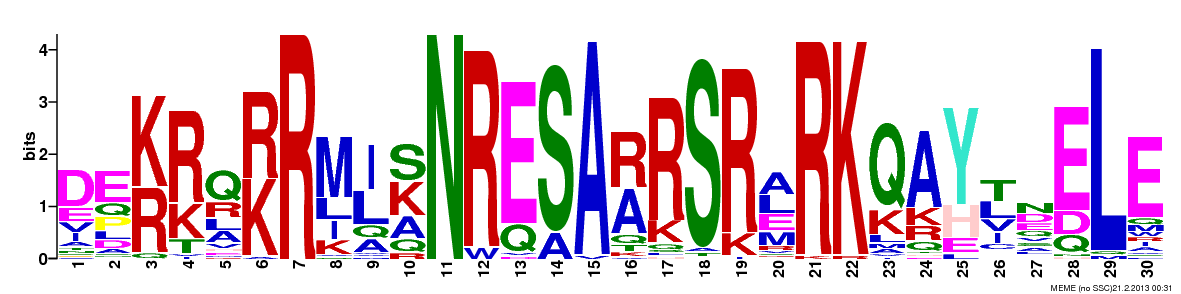 |
| **Motif 2** | **7.2e-420** | GD[GQ][AFS][HS][GS]M[IS][GNS][NS]G[AI][AL]AF[DE][MV]EY[AG][RH]W[LV]EE[HQ][HN]R[QL][IM][CN]ELR[ST]A[VL][NQ][AS]H[ALV]SDN[ED]LR[IV][IL]V[DE][NG][AV][ML][AN]H[YF]D[ED][IL]FR[LM]K[GAS][MV]AAK[AS]DVF[HY][LM][LM]SGMWKT[PS]AER[CF]F[LM]W[ILM]GGFR[PS]SE[LV][LI]K[ILV]LV[NP]QL[ED]PLT[ED]QQ[LI][LM][GD][IV][CY][NG]L[QR]QSSQQAE[DE]ALSQGME[AK]LQQSLA[ED]T[IL][AS]S[GD]S[LV]G  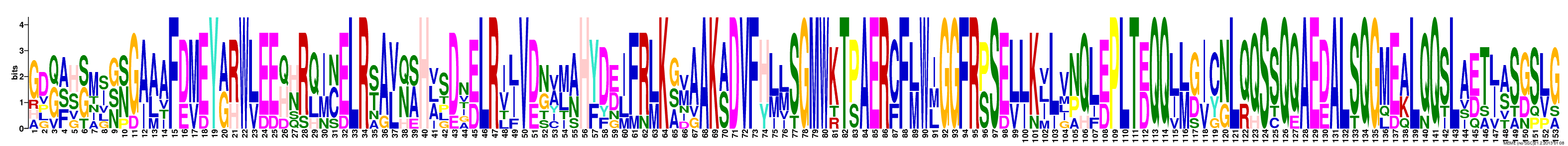 |
| **Motif 3** | **3.0e-196** | [RH][KS]V[QT][TS]LQ[TA]E[AGV][TS][TE]LS[AP][QR][LV][TA][LF]L[QD][RHQ][DQ][TNRS][LTA][GI]L[TNS][AMSTV][ED]N[SKNT][EA]LK[LQ]R[LI][QAE][AS][ML][EA]Q[QED][AK][QIL][LFI][RK]D[AL][LEH][NQ][ED]AL[KET][REK]E[IV][EQ]RL[KRQ][ILQ][ALV][TY][GH][QE][QI][MPQ]  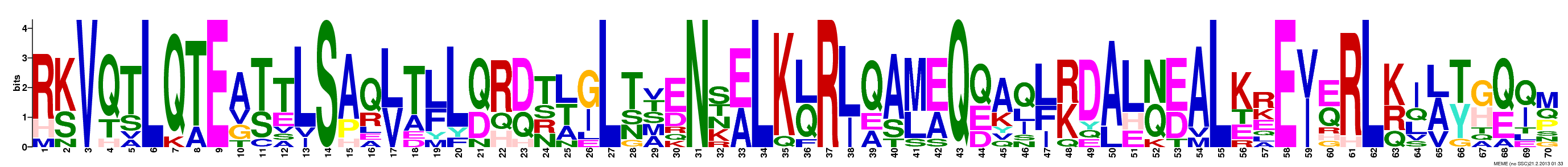 |
| **Motif 4** | **3.6e-161** | LG[TA]LE[GS]F[VL][RN]QAD[NH]LRQQT[LI][QH]Q[ML]HRILT[TV]RQ[AS]AR[ACG]LL[AV][IL]G[ED]YF[SH]RLRALSSLW[AL]ARPR  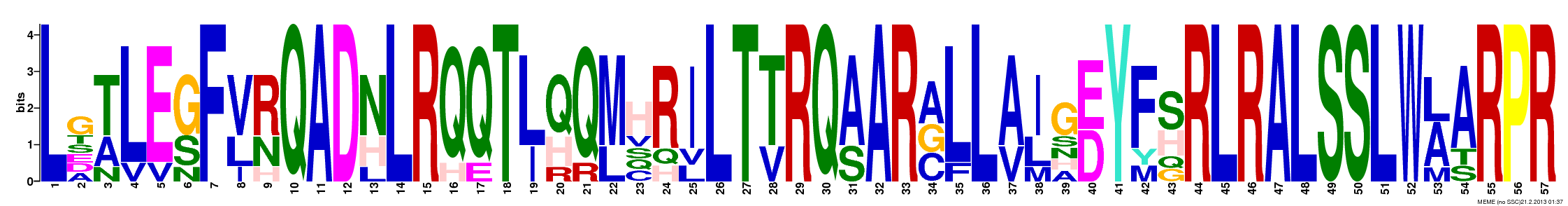 |
| **Motif 5** | **5.1e-100** | x[EKQ]V[EAS][QR]L[RK]EENxxL[RK]K[RK]LxxL  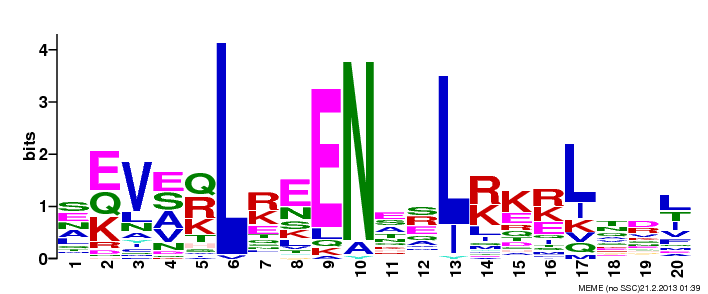 |
| **Motif 6** | **5.7e-072** | [FN]Q[PS]L[AV]RQ[GNS]S[IM]YSLT[LF]DE[FV]QN[QT]LG[DG][LV]GK[PD][LF][GT]SMN[LM]DELL  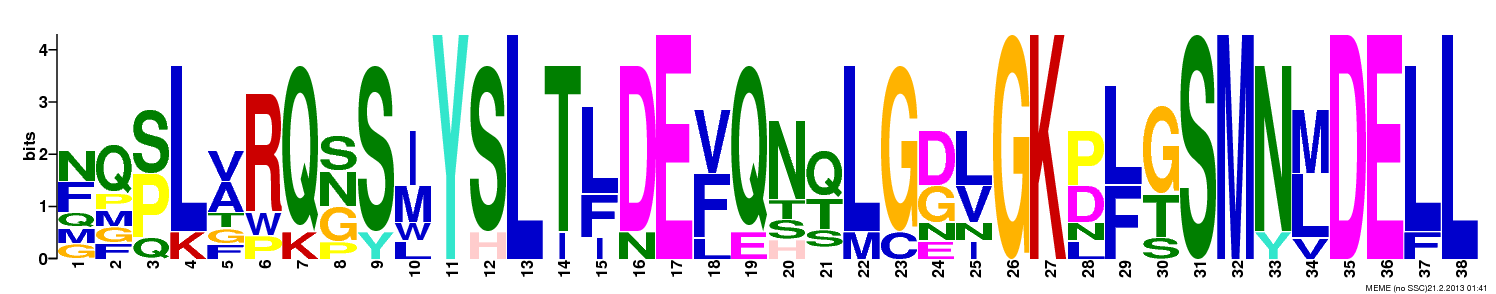 |
| **Motif 7** | **9.4e-071** | [QE]R[QE]PTLGEMTLE[DE]FL[VA][KR]AG[VA]V[RA]E  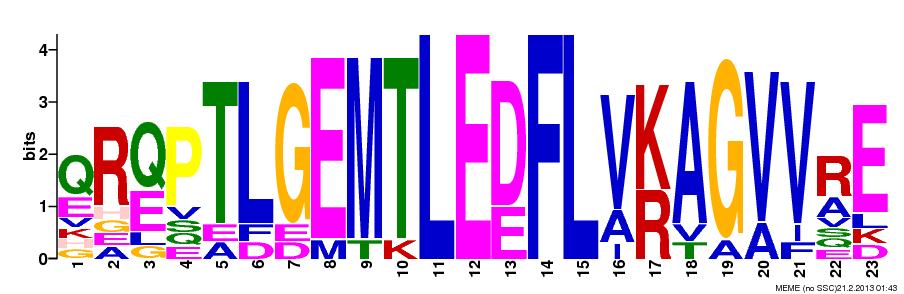 |
| **Motif 8** | **2.8e-052** | [GA]S[LF][ST]LPG[AT]LS[KQ]KTV[DE]EVW[KS][DE]I  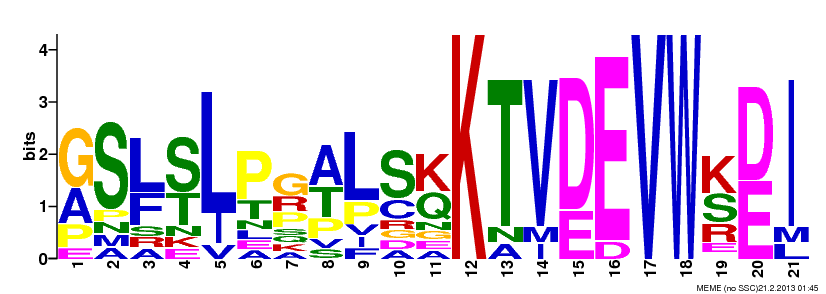 |
| **Motif 9** | **9.3e-047** | R[ILM]KLTQLEQEL[QE]RARQQG[LI][FY]I  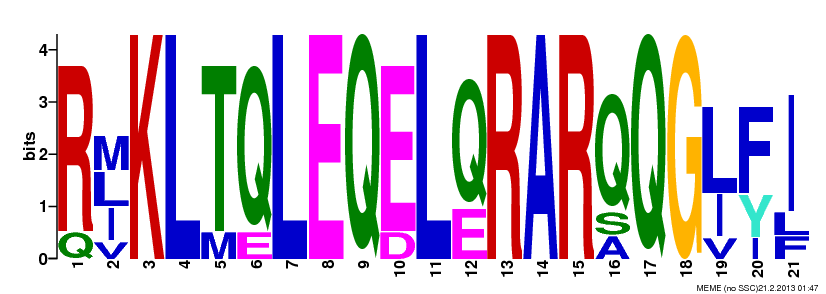 |
| **Motif 10** | **9.1e-024** | [PA][HY]PY[LIMV]W[GA][PGSV][QPS][HQ][PL][ML][IMP]P[PY][YG][GT]  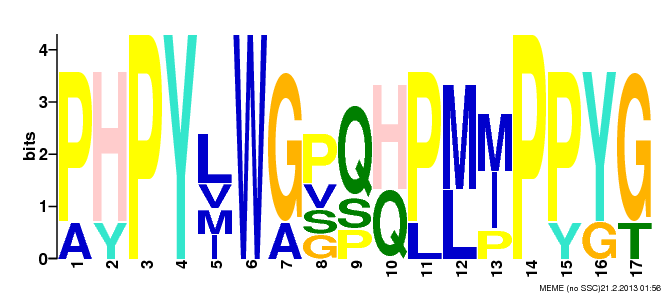 |
| **Motif 11** | **5.0e-030** | [TN]QKYN[NE][AIV][AE][ASV][DE]N[NRS][VI]L[RK]A[QD]V[EM][ET]L[RST]D[RK][LV]Q[SM][LA][NE][ED]I[LV][KQ][RY][VL][NT][GT][SLV]NG[LV]  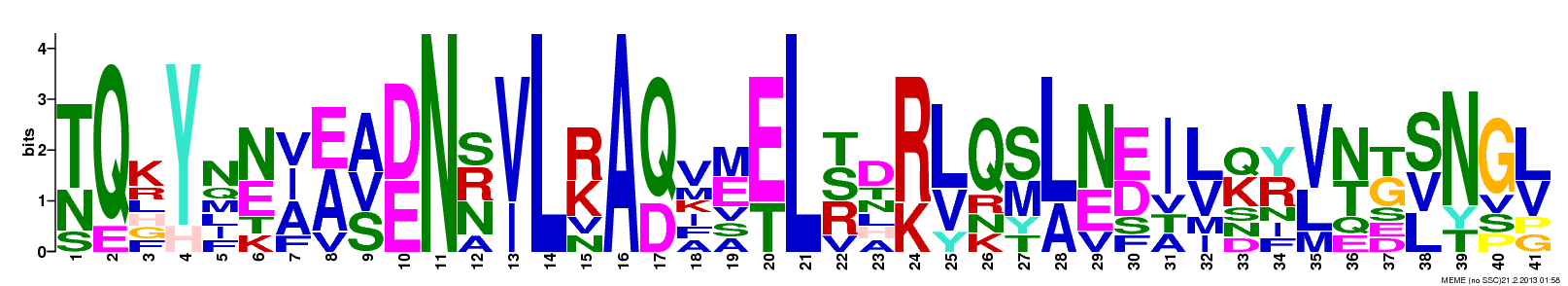 |
| **Motif 12** | **9.1e-029** | DKKI[AD]GTVIGVDPNV[GR]PQFPQQGQWMQYPQPQFPHPQQNM[IK]GVYMPGQPMPQPLPMGPSSVMDV[IT]YPDNQVALSSPLMGA  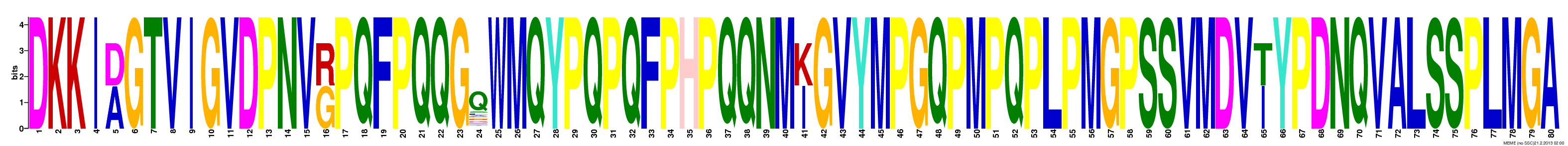 |
| **Motif 13** | **4.2e-020** | PYxA[IL]Y[PS][PH]GG[VL]Y[AP]HP  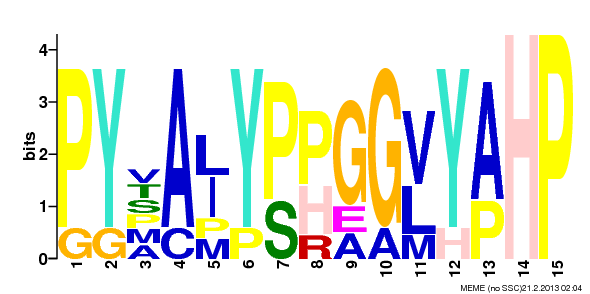 |
| **Motif 14** | **6.0e-018** | N[VM]A[ND]YMGQMA[IM]AM[GN]K  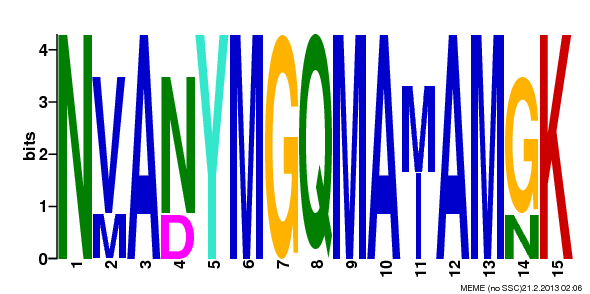 |
| **Motif 15** | **1.9e-017** | [NMT]W[GR][ED][ST][FAGN][MK][AGT][DN][AGNS][SCN][PQ][RINQ]T[DGS][ATI]ST[DI][VAM][DEQ][TLV][DE][DAET][KM][LNV][QD][NRY][FHKQ]  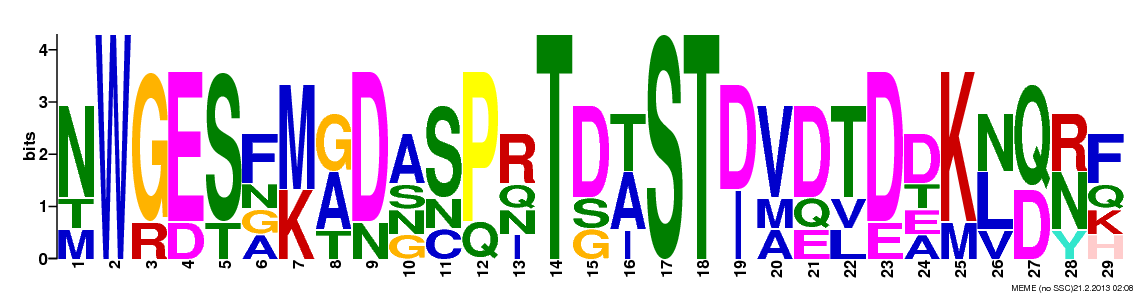 |
| **Motif 16** | **4.3e-017** | [CT][PS][IM]DSF[FL]D[DE][FI]LK[DN]T[HR][AT]CTHTHTCNPPGPD[AF][AS]HTHTC[FY]H[TV]HT  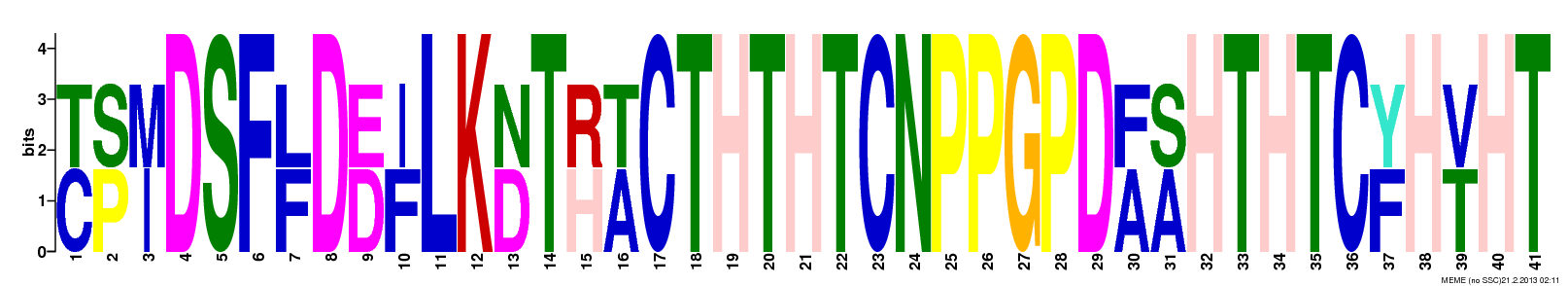 |
| **Motif 17** | **3.1e-015** | QQQQQQQ[QH][HQ]QQ  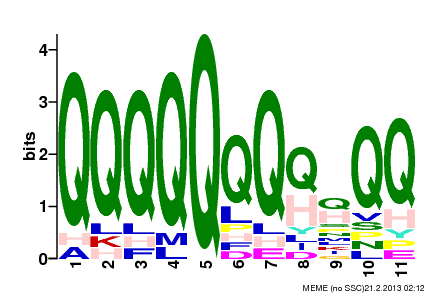 |
| **Motif 18** | **1.7e-013** | KK[AI]M[AG][PN][DE]KLAE[LI][AW]LI  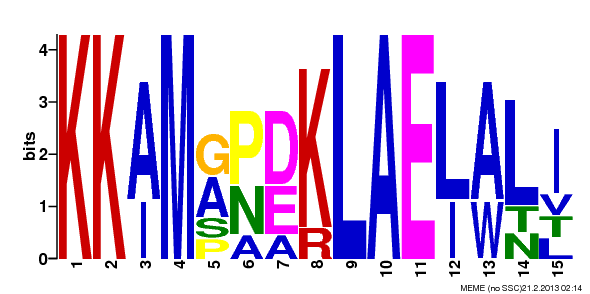 |
| **Motif 19** | **2.0e-010** | [HG]HRR[SA]SS[DE][SD]L[AF]  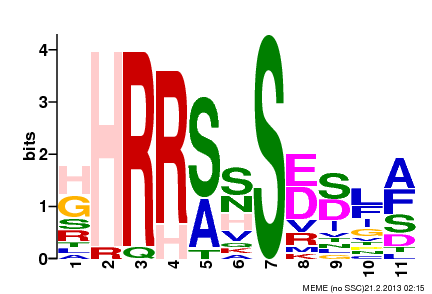 |
| **Motif 20** | **2.6e-009** | [RY][QCTV]LRR[TV][SL][ST][AGV][PL][FW]  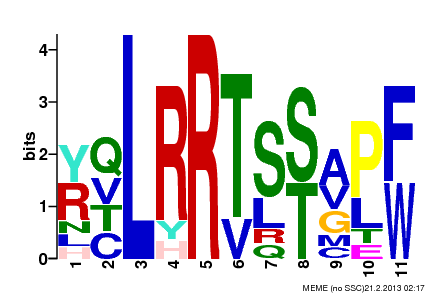 |
