# Supplementary material for: Genome-wide analysis and expression profile of the bZIP transcription factor gene family in grapevine (Vitis vinifera)
Source: BMC Genomics. 2014 Apr 13;15:281. doi: 10.1186/1471-2164-15-281 (PMC4023599; doi:10.1186/1471-2164-15-281)
Supplement: Additional file 4 — The length of Leucine zipper region in bZIP domain of VvbZIP proteins. [file 1471-2164-15-281-S4.pdf]

Additional file 4. The length of Leucine zipper region in bZIP domain of VvbZIP proteins

```
VvbZIP08   VVERRQRRMIKNRESAARSRARKQAYTLELEMEVAKLKEANEEL
VvbZIP45   VIERRQRRMIKNRESAARSRARKQAYTMELEAEVAKLKEKNEEL
VvbZIP34   TVERRQKRMIKNRESAARSRARKQAYTNELENKVSRLEEENERL
VvbZIP31   TIERRQKRMIKNWESATHSRARKQAYTNELENKVSRLEEENERL
VvbZIP18   TVERRLKRKIKNRESAARSRARKQAYHNELVSKVSRLEEENVRL
VvbZIP43   TIERRQKRMIKNRESAARSRARKQAYTNHLEHEVHQLKKENDLL
VvbZIP19   AVERRQRRMIKNRESAARSRARKQAYTVELELELNQLKEENTKL
VvbZIP25   VVERRQRRMIKNRESAARSRARKQAYTVELEAELNQLKEENTLL                                                     I
VvbZIP30   ATQQRQRRMIKNRESAARSRERKQAYTVELESLVTHLEEENARL
VvbZIP51   AAQQKQRRMIKNRESAARSRERKQAYQVELESSAVRLEEENEQL
VvbZIP49   SGDRRHKRLIKNRESAARSRARKQAYTNELELEVAHLIEENARL
VvbZIP11   TIERRQKRMIKNWESATRSRARKQAYTNELENKVSRLEEENERL
VvbZIP06   AEKKSKKRPLGNREAVRKYREKKKARAASLEDEVVRLRSLNQQL
VvbZIP36   HLISKVRRPSGNREAVRKYREKKKAHTAYLEEEVKKLRLLNQQL
VvbZIP24   EEEKKKARLMRNRESAQLSRQRKKHYVEELEEKIRSMHSTIQDL

VvbZIP27   GDQKTLRRLAQNREAARKSRLRKKAYVQQLENSRMKLTQLEQELQRARQQG
VvbZIP35   -DQKTLRRLAQNREAARKSRLRKKAYVQQLESSRMKLTQLEQELQRARQQG
VvbZIP01   GDQKTLRRLAQNREAARKSRLRKKAYVQQLECSQLKLTQLEQELQRARQQG
VvbZIP23   PVDKVQRRLAQNREAARKSRLRKKAYVQELESSRVKLMQLEQELERARQQG                                      II
VvbZIP42   TTDKTQRRLAQNREAARKSRLRKKAYVQQLETSRLKLTELEQELERARQQG
VvbZIP28   PDPKTLRRLAQNREAARKSRLRKKAYVQQLESSRIKLTQLEQELQRARAQG

VvbZIP53   AAEKIRRRMIKNRESAARSRARKLAYDAQQQIEIAKLKKENEFLRRIIRVL                                      III
VvbZIP03   RQLKRERRKQANRESAKKSRLRKQAENEELRMRYETLNEENKALKFEISKLTEHLDKVRLENTAL

VvbZIP12   RELKRQKRKQSNRESARRSRLRKQAECEELQAKVETLSTENTALRDELQRLSEECEKL
VvbZIP46   RELKRQRRKQSNRESARRSRLRKQAECEELQSKVEILSNENHVLREELHRLAEQCEKL                               IV
VvbZIP15   IKLVLESRLLRNRVSAQQARERKKVYVNDLESRAQELQDRNSKLEEKISTLVNENTML
VvbZIP10   KENKRLKRLLRNRVSAQQARERKKAYLNELEVRVKDLERKNSELEERLSTLQNENQML

VvbZIP40   REIKRERRKQSNRESARRSRLRKQAETEELALKVESLNTENSVLKSEINRLRENSEKLKLENATL
VvbZIP05   TDPKRAKRILANRQSAARSKERKMRYISELEHKVQTLQTEATTLSAQLTLLQRDSAGLTSQNNELKFRLQAMEQQAQLRDALNEALTAEVQRL
VvbZIP48   VDPKRAKRILANRQSAARSKERKVRYMAELEHKVHTLQTETTTLSHLLTLLQRDSAELTSRNNELKLRIQAMEQEAQFRDALKEALTLEVHRL     V
VvbZIP50   IDPKRAKRILANRQSAARSKERKIRYTNELERKVQTLQTEATTLSAQVTMLQRDTTGLTAENKELKLRLQAMEQQASLREALNEALREEVQRL
VvbZIP16   IDPKRAKRIWANRQSAARSKERKMRYIAELERKVQTLQTEATSLSAQLTLLQRDTNGLTAENSELKLRLQTMEQQVNLQDALNDALKEEIQHL

VvbZIP29   VDPKRVKRILANRQSAQRSRVRKLQYISELERSVTSLQTEVSVLSPRVAFLDHQRLLLNVDNSALKQRIAALAQDKIFKDAHQDALKREIERL     VI
VvbZIP52   IDPKRVKRILANRQSAQRSRVRKLQYISELERSVTSLQTEVSALSPRVAFLDHQRLILNVDNSALKQRIAALAQDKIFKDAHQEALKKEIERL

VvbZIP41   QASKKRRRQLRNRDAAVRSRERKKTYVRDLELKSRYLESECRRLGHLLQCCFAENQTL
VvbZIP04   RELKRQRRKQSNRESARRSRLRKQAECDELAQRADALKEENASLRAEVSRIKSEYEQLLSENASL
VvbZIP26   KEARRLRRVLANRESARQTIRRRQALCGELSRKAADLSLENETLKREKELAMKEFQSLENKNKHL
VvbZIP17   IDPKRAKRILANRQSAARSKERKARYILELERKVQTLQTEATTLSAQLTLYQRDTTGLSTENTELKLRLQAMEQQAQLRDALNTALKQEVERL     VII
VvbZIP33   LDPKRAKRILANRQSAARSKERKARYILELERKVQTLQTEATTLSAQLTLFQRDTTGLTTENTELKLRLQAMEQQAQLRDALNEALKKEVERL

VvbZIP55   AEERKRRRMISNRESARRSRMRKQKHIENLRNQLNQLRIQNRELTNRLRSFTYHSHLVDSDNVQLRSEAIIL
VvbZIP14   VDEKKRKRMISNRESARRSRMRKQQHLDDLIKRKSELENQRLEIKRRIDMFQKLWEATVGENNALEALKAEL
VvbZIP21   ADEKRARRMLSNRESARRSRRRKQEHLSELETQVSQLGVENSSLLKRLTDINQKYNEAAVDNRVLKADVETL                         VIII
VvbZIP38   ADAKRVRRMLSNRESARRSRRRKQAHLTELETQVAQLRLENSSLLKRLTDISQKYNEAAVDNRVLKADVETL
VvbZIP09   NNLKRMRRMVSNRESARRSRKRKQAHLADLELQVEQLRGENASLYKQLTDASQQFGDANTNNRVLKSDVEAL

VvbZIP02   IDERKQRRMISNRESARRSRMRKQKHLDELWSQVVRLRNENHSLIDKLNHVSECHDRVLQENVRLKEEASDLRQMLTDLRIGSPYTTLRELELRQMLTDL
VvbZIP39   INERKQRRMISNRESARRSRMRKQKHLDELWSQVVWLRNENHQLIDKLNHVSECHDRVLQENVQLKEEASELRQMVTDLQLNSPYPNLRDLELRQMVTDL
VvbZIP44   --------MISNRESARRSRWRKKKHLENLSNEVNRLLVQNREYKHRLGSVTHQCHLVGRDNERLTYEYLALRTKLYDL
VvbZIP07   MDQRKRKRMISNRESARRSRMRKQKHLDDLMAQAAQLRKENSQIITSMNVTTQHYFNIEAENSVLRAQFSELSNRLQYLVEIISFL
VvbZIP13   MDQRKRKRMLSNRESARRSRMRKQKHLDDMMAQMVHLRKENNRILTTMNVTTQFHMNVEAENAILRAQMAELTLRLQTLNEIMDYL           IX
VvbZIP47   MDQRKRKRMLSNRESARRSRMRKQKHLDDLMAQVAQLRKENNEILSSINITNQRYLTVEADNSILRAQAMELSHRYQSL
VvbZIP22   IDDRKRKRMQSNRESAKRSRIRKQQHLDDLLSKAAQLQKENGQIAERIDKTTELYIKIASDNNVLNAQIVELTDRLQSL
VvbZIP37   MDEKKRRRMLSNRESARRSRMKKQKLSEDLISEVSRLQNLNKEIKQTIDATTQGYQNFVSENNVLVAQKMELVDRLNSL

VvbZIP32   ETDTKRAKQQFAQRSRVRKLQYIAELERNVQALKAEGSEVSAELDFLNQQNLILSMENKA                                     U
VvbZIP54   KNDAKRAKQQFAQRSRLRKLQYIAELEMSVQVLQAEGCEISAAVEYLDQHNLILGMKNRA
```
