# Supplementary material for: Genome-wide analysis and expression profile of the bZIP transcription factor gene family in grapevine (Vitis vinifera)
Source: BMC Genomics. 2014 Apr 13;15:281. doi: 10.1186/1471-2164-15-281 (PMC4023599; doi:10.1186/1471-2164-15-281)

Additional file 5. Alignment of bZIP domain of VvbZIP proteins.

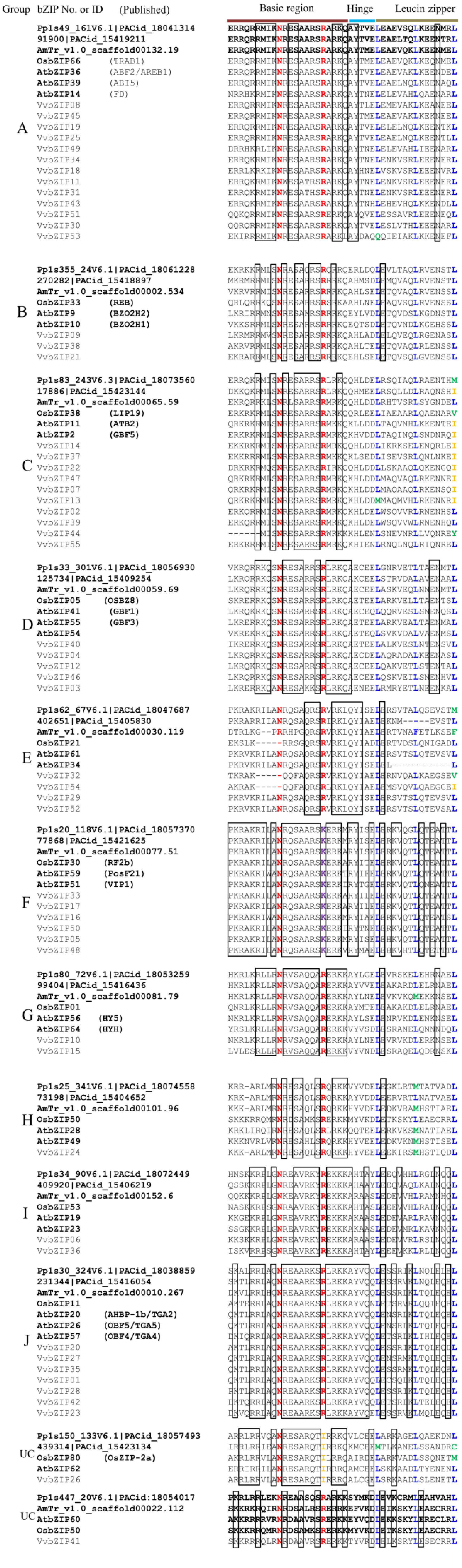

Supplement: Additional file 5 — Alignment of bZIP domain of VvbZIP proteins. [file 1471-2164-15-281-S5.pdf]
