# Supplementary material for: Genome-wide analysis and expression profile of the bZIP transcription factor gene family in grapevine (Vitis vinifera)
Source: BMC Genomics. 2014 Apr 13;15:281. doi: 10.1186/1471-2164-15-281 (PMC4023599; doi:10.1186/1471-2164-15-281)
Supplement: Additional file 6 — Description of organs and tissues of Vitis vinifera cultivar Corvina, from a vineyard in the Verona province (Montorio). [file 1471-2164-15-281-S6.docx]

**Additional file 6.** Description of organs and tissues of *Vitis vinifera* cultivar Corvina, from a vineyard in the Verona province (Montorio).

| Organ | Development stages collected (54 samples) | Abbreviations | E-L  system stages |
| --- | --- | --- | --- |
| Inflorescance | Young inflorescence (single flower in tips visible) | Inflorescence-Y | E-L 14 |
|  | Well developed inflorescance (single flower separated) | Inflorescence-WD | E-L 17 |
| Flower | Flower begins (10% caps off) | Flower-FB | E-L 20 |
|  | Flowering (50% caps off) | Flower-F | E-L 23 |
| Stamen | Pool of stamens from undisclosed flowers at 10% and 50% open flowers | Stamen | E-L 20/ E-L 23 |
| Petal | Pool of petals from undisclosed flowers at 10% and 50% open flowers | Petal | E-L 20/E-L 23 |
| Pollen | Pollen from disclosed flowers at more than 50% open flowers | Pollen | E-L 25 |
| Carpel | Pool of carpels from undisclosed flowers at 10% and 50% open flowers | Carpel | E-L 20/E-L 23 |
| Berry Pericarp | Fruit set | Berry Pericarp-FS | E-L 29 |
|  | Post- fruit set | Berry Pericarp-FFS | E-L 32 |
|  | veraison | Berry Pericarp-V | E-L 35 |
|  | Mid-ripening | Berry Pericarp-MR | E-L 36 |
|  | Ripening | Berry Pericarp-R | E-L 38 |
|  | Post-harvest witheringⅠ(1^st^ month) | Berry Pericarp-PHWⅠ |  |
|  | Post-harvest witheringⅡ(2^nd^ month) | Berry Pericarp-PHWⅡ |  |
|  | Post-harvest witheringⅢ (3^rd^ month) | Berry Pericarp-PHWⅢ |  |
| Berry Flesh | Post fruit set | Berry Flesh-FFS | E-L 32 |
|  | Veraison | Berry Flesh-V | E-L 35 |
|  | Mid-ripening | Berry Flesh-MR | E-L 36 |
|  | Ripening | Berry Flesh-R | E-L 38 |
|  | Post-harvest witheringⅠ(1^st^ month) | Berry Flesh-PHWⅠ |  |
|  | Post-harvest witheringⅡ(2^nd^ month) | Berry Flesh-PHWⅡ |  |
|  | Post-harvest witheringⅢ (3^rd^ month) | Berry Flesh-PHWⅢ |  |
| Berry Skin | Post-fruit set | Berry Skin-FFS | E-L 32 |
|  | Veraison | Berry Skin-V | E-L 35 |
|  | Mid-ripening | Berry Skin-MR | E-L 36 |
|  | Ripening | Berry Skin-R | E-L 38 |
|  | Post-harvest witheringⅠ(1^st^ month) | Berry Skin-PHWⅠ |  |
|  | Post-harvest witheringⅡ(2^nd^ month) | Berry Skin-PHWⅡ |  |
|  | Post-harvest witheringⅢ (3^rd^ month) | Berry Skin-PHWⅢ |  |
| Seed | Fruit set | Seed-FS | E-L 29 |
|  | Post-fruit set | Seed-FFS | E-L 32 |
|  | Veraison | Seed-V | E-L 35 |
|  | Mid-ripening | Seed-MR | E-L 36 |
| Rachis | Fruit set | Rachis-FS | E-L 29 |
|  | Post-fruit set | Rachis-FFS | E-L 32 |
|  | Veraison | Rachis-V | E-L 35 |
|  | Mid-ripening | Rachis-MR | E-L 36 |
|  | Ripening | Rachis-R | E-L 38 |
| Tendril | Young tendril (pool of tendrils from shoot of 7 leaves) | Tendril-Y | E-L 14 |
|  | Well developed tendris (pool of tendrils from shoot of 12 leaves) | Tendril-WD | E-L 17 |
|  | Mature tendril (pool of tendrils at fruit set) | Tendril-FS | E-L 29 |
| Leaf | Young leaf (pool of leaves from shoot of 5 leaves) | Leaf-Y | E-L 14 |
|  | Mature leaf (pool of leaves from shoot at fruit set) | Leaf-FS | E-L 29 |
|  | Senescencing leaf (pool of leaves at the beginning of leaf fall) | Leaf-S | E-L 43 |
| Stem | Green stem | Stem-G | E-L 14 |
|  | Woody stem | Stem-W | E-L 43 |
| Bud | Latent bud | Bud-L | E-L 23 |
|  | Witer bud | Bud-W | E-L 1 |
|  | Bud swell | Bud-S | E-L 2 |
|  | Bud burst (green tip) | Bud-B | E-L 4 |
|  | Bud after-burst (rosette of leaf tips visible) | Bud-AB | E-L 5 |
| Seedling | Pool of 3 developmental stages | Seedling |  |
| Root | *In vitro* cultivation | Root |  |

Note: E-L, classiﬁcation key of tissues and organ developmental stage deriving from the original paper [[1](#_ENREF_1)].

**Refrence**

1. Fasoli M, Dal Santo S, Zenoni S, Tornielli GB, Farina L, Zamboni A, Porceddu A, Venturini L, Bicego M, Murino V *et al*: **The grapevine expression atlas reveals a deep transcriptome shift driving the entire plant into a maturation program**. *The Plant cell* 2012, **24**(9):3489-3505.
