# Supplementary figures and images for: Genome-wide analysis and expression profile of the bZIP transcription factor gene family in grapevine (Vitis vinifera)
Source: BMC Genomics. 2014 Apr 13;15:281. doi: 10.1186/1471-2164-15-281 (PMC4023599; doi:10.1186/1471-2164-15-281)

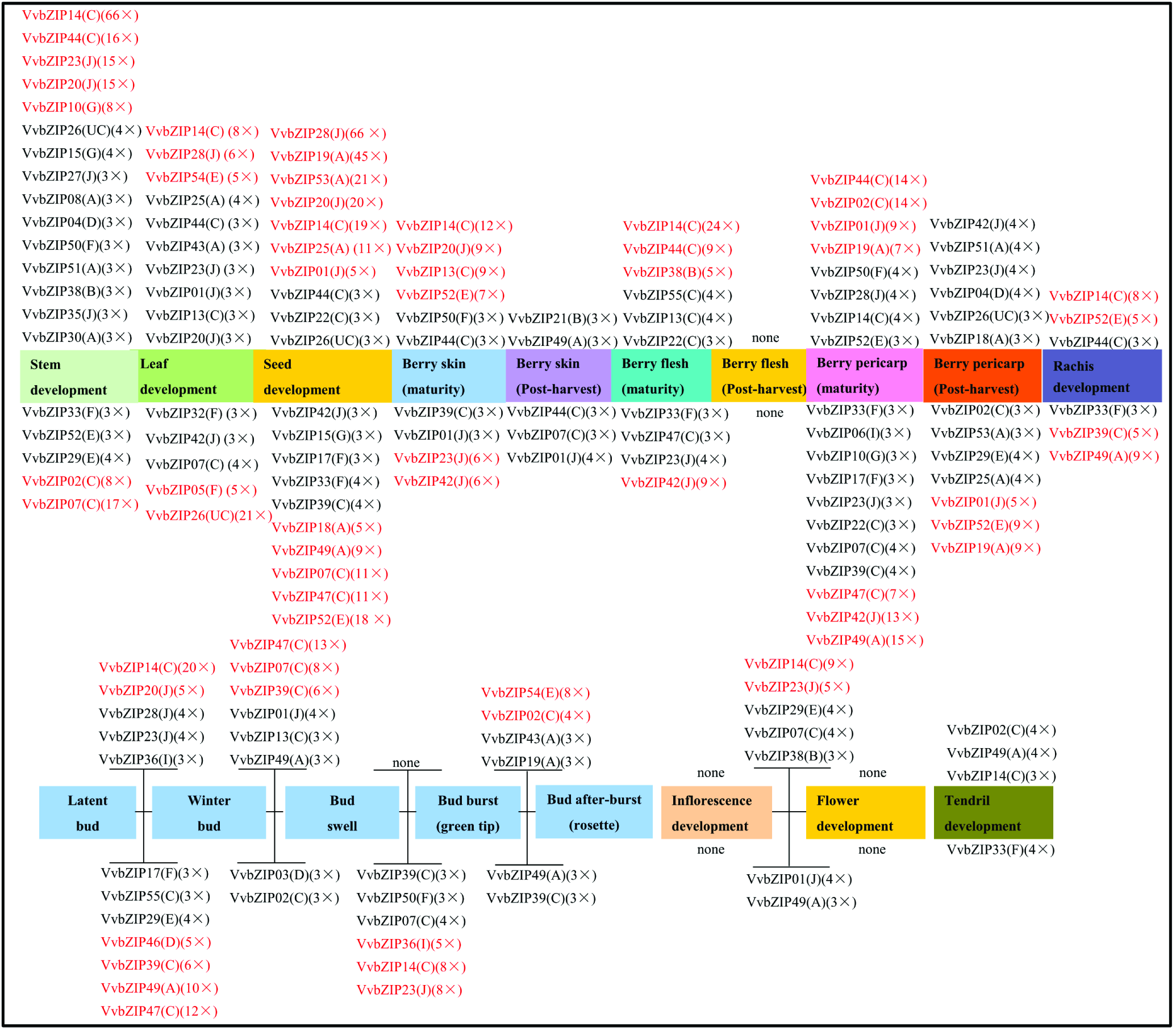

Supplement: Additional file 8 — Differentially expressed genes of grapevine bZIPs in various kinds of tissues. The microarray data was normalized based on setting up the expression level of first development stage of each tissue as 1, the folds of differential expression was subsequently calculated. The genes above and below the colored tissue bar indicated the up-regulated and down-regulated genes during this tissue development, respectively. The expression fold was presented at bracket along with each VvbZIP gene. The genes fluctuated more than 5 folds was highlight with red characters. [file 1471-2164-15-281-S8.tiff]
